# Comparative Analysis of the PYL Gene Family in Three *Ipomoea* Species and the Expression Profiling of *IbPYL* Genes during Abiotic Stress Response in Sweetpotato

**DOI:** 10.3390/genes14071471

**Published:** 2023-07-19

**Authors:** Lei Zhang, Weihan Song, Guosheng Xin, Mingku Zhu, Xiaoqing Meng

**Affiliations:** 1 Yantai Academy of Agricultural Sciences, Yantai 261417, China; kerqzl@126.com (L.Z.); guoshengx@sina.com (G.X.); 2 Jiangsu Xuzhou Sweetpotato Research Center, Xuzhou 221131, China; xzsongweihan@126.com; 3 Institute of Integrative Plant Biology, School of Life Sciences, Jiangsu Normal University, Xuzhou 221116, China

**Keywords:** ABA receptor, abiotic stress, gene expression, molecular characterization, sweetpotato

## Abstract

Abscisic acid (ABA), a critical phytohormone that regulates plant development and stress response, is sensed by the ABA receptors PYR/PYL/RCAR (PYLs). The PYL genes have been widely studied in multiple plant species, while a systematic analysis of PYL genes in the genus *Ipomoea* remains unperformed. Here, a total of 13, 14, and 14 PYLs were identified in *Ipomoea batatas*, *Ipomoea trifida*, and *Ipomoea triloba*, respectively. Fragment duplication was speculated to play prominent roles in *Ipomoea* PYL gene expansions. These *Ipomoea* PYLs were classified into three subfamilies via phylogenetic analysis, which was supported by exon–intron structures and conserved motif analyses. Additionally, the interspecies collinearity analysis further depicted a potential evolutionary relationship between them. Moreover, qRT-PCR analysis showed that multiple *IbPYLs* are highly and differentially responsive to abiotic stress treatments, suggesting their potential roles in sweetpotato stress responses. Taken together, these data provide valuable insights into the PYLs in the genus *Ipomoea*, which may be useful for their further functional analysis of their defense against environmental changes.

## 1. Introduction

Due to their immovable lifestyle, harsh environmental conditions, such as salinity and drought, significantly affect the growth of plants and reduce the yield of crops [1,2]. To survive in harsh environments, plants have evolved diverse defense mechanisms involving many processes, such as perception, signal transduction, transcriptional processing, translation, and post-translational modification [3,4]. Abscisic acid (ABA) not only plays important roles in the diverse growth and development of plants, but also imparts cells with tolerance to multiple biotic and abiotic stresses [1,5,6]. Recent work has shown that ABA is perceived by ABA receptor PYR/PYL/RCAR (PYLs), and the binding enables it to interact with clade A type 2C protein phosphatases (PP2Cs), which in turn inhibits the activity of PP2Cs and thus relieves sucrose nonfermenting 1-related protein kinase 2 (SnRK2s) to activate downstream targets and promote the physiological response to ABA [7,8,9].

As the core regulator in ABA signaling, substantial efforts have been made to characterize the PYL gene family from Arabidopsis, which encodes 14 AtPYLs that are highly conserved in sequences and functional domains [9,10]. To date, PYL members have been widely identified at genome-wide levels in diverse plant species, such as tobacco [11], rice [12], cotton [13], cucumber [14], wheat [15], and *Brassica napus* [11]. Massive studies have shown that AtPYLs play diverse roles in development and stress response. AtPYR1, AtPYL-1/-2, AtPYL-4/-5, AtPYL-8/-9, and AtPYL13 can promote ABA-induced seed germination, stomata closure, and root growth; AtPYL5, AtPYL9, and AtPYL13 can provide drought tolerance [12,16,17]. And rice OsPYL-2/-8/-9/-10/-PYL11 were also functionally characterized to affect abiotic stress tolerance or yield [12,18]. However, the functional revelation of most PYLs from many plants in development and stress response remains to be investigated.

There are 500–600 species of genus *Ipomoea*, which is the most prevalent species in the Convolvulaceae family, and *Ipomoea* species are widely used with great value in industry and agriculture [19]. Sweetpotato (*Ipomoea batatas*, 2n = 6x = 90) is the seventh-most-important crop worldwide because of its strong adaptability, stable yields, high starch content, strong stress resistance, and low input requirement [20,21]. To date, the genomes of sweetpotato, *I. trifida*, and *I. triloba* have been sequenced, and *I. trifida* is identified as the most closely related diploid to sweetpotato, followed by *I. triloba* [22,23]. Recently, IbPYL8-IbbHLH66-IbbHLH118 complex was shown to mediate the ABA-dependent drought response in sweetpotato [24]. Nevertheless, a genome-wide comparative analysis of PYL genes in *Ipomoea* is still lacking.

In this study, a genome-wide systematical analysis of PYL genes was conducted in three *Ipomoea* species, including *I. batatas*, *I. trifida*, and *I. triloba*, including their chromosomal locations, phylogenetic relationships, gene duplications, gene structures, conserved domains, and the response to abiotic stress. This work provides valuable insights into the PYLs in the genus *Ipomoea*, which may be useful for their further functional analysis in defense environmental changes.

## 2. Materials and Methods

### 2.1. The Identification of Putative PYL Genes in Three Ipomoea Species

The genome sequences and GFF profiles of three *Ipomoea* species including *I. batatas*, *I. trifida*, and *I. triloba* were downloaded from the *Ipomoea* Genome Hub database [22] and Sweetpotato Genomic Resource [23]. PYL genes in the three *Ipomoea* species were identified using the reported protein sequences of six plant species, including 14 *Arabidopsis* AtPYLs, 13 rice OsPYLs, 38 wheat TaPYLs, 13 maize ZmPYLs, 9 barley HvPYL, and 8 sorghum SbPYLs (Table S1), as found by searching the protein databases of three *Ipomoea* species using a BLASTP program through a default parameter. All the candidate PYLs were detected using NCBI Batch CD-Search and ScanProsite programs. The sequences of PYLs in the three *Ipomoea* species are presented in Table S2.

### 2.2. Chromosomal Distribution and Genome-Wide Syntenic Analysis of PYL Genes

The chromosomal distribution of PYL genes in three *Ipomoea* species was determined with respect to their starting and ending position information retrieved from each GFF profile. The tandem duplication and segmental duplication of PYL genes in three *Ipomoea* species were accomplished by MCScanX software v0.8.25 [25]. An analysis of homologous gene pairs of PYLs among sweetpotato, *I. triloba*, *I. triloba*, Arabidopsis, rice, tomato, pepper, cabbage, and *Brassica oleracea* was determined with MCScanX using default parameters. The results were visualized using Circos and TBtools software v1.120, and the block sizes were set to 30 [26,27,28].

### 2.3. The Analysis of Gene Structures, Conserved Motifs, and Protein Properties

The molecular weights (kDa) and isoelectric points (pIs) of each PYL were predicted using the ExPASy database, and phosphorylation sites were investigated using the NetPhos 3.1 Server both with a default parameter. The gene structures of PYLs were visualized using with information in the TBtools software v1.120 [26]. The conserved motifs of PYLs were detected by MEME 5.5.2 using the parameter reported in rice [12,29].

### 2.4. The Phylogenetic Classification of Three Ipomoea PYLs

For the phylogenetic classification of three *Ipomoea* PYLs, the complete amino acid sequences of PYL proteins of *Arabidopsis*, *I. batatas*, *I. trifida*, and *I. triloba* were analyzed. Multiple sequence alignment was conducted with the ClustalW program using a default parameter. An unrooted phylogenetic tree was performed using the best model WAG + G via the MEGA software (X version) through the maximum likelihood method [30], and a bootstrap value of 1000 was adopted.

### 2.5. RNA Extraction and qRT-PCR Analysis

For abiotic stress treatments, the detailed process for salt and drought stress treatments was described before [31]. Total RNAs were extracted using RNA extraction kits (Tian Gen, Beijing, China), 1 μg RNA of each sample was reverse-transcribed by TransScript one-step gDNA removal and cDNA synthesis mix (TransGen, Beijing, China). The qRT-PCR experiment was conducted on a CFX96^TM^ real-time system (Bio-Rad, Hercules, CA, USA) with the processes described previously [31], and the sweetpotato *ARF* gene (JX177359) was applied as an internal reference [32]. The primers for qRT-PCR analysis are found in Table S3.

### 2.6. Statistical Analysis

To rigorously screen for the stress-induced *IbPYLs*, a cut-off value of two-fold was adopted [33].

## 3. Results

### 3.1. The Identification and Chromosomal Location Analysis of PYLs in Three Ipomoea Species

The BLASTP program was used to identify all the putative PYL genes in the the *Ipomoea* species, and the NCBI Batch CD-Search and ScanProsite programs were employed to validate the obtained results. A total of 13, 14, and 14 PYL genes were identified in the genome databases of *I. batatas* (Ib), *I. trifida* (Itf), and *I. triloba* (Itb), respectively. The Ipomoea SPL genes were named *IbPYL1*~*IbPYL13L*, *ItfPYL1*~ *ItfPYL14*, and *ItbPYL1*~*ItbPYL14*, respectively, based on their chromosomal locations. It should be noted that two *PYL-likes* were identified in *I. batatas* genomes because of their atypical sequence composition; thus, 11 *IbPYL* genes were named successively, while 2 unique *IbPYLs* were then named ignoring the order of their chromosomal locations (Figure S1). Moreover, although two transcripts of itb05g20040 were detected, itb05g20040.t1 (*ItbPYL11.1*) and itb05g20040.t2 (*ItbPYL11.2*), they encoded completely identical amino acid sequences, so only ItbPYL11.1 was included in the downstream analysis.

The chromosomal location analysis displayed that PYL genes were unevenly distributed in the chromosomes of three *Ipomoea* species, of which the Chr12 chromosome of *I. trifida* and *I. triloba* both possess the most abundant PYL genes, with each containing four PYL genes, followed by the LG5 chromosome of *I. batatas,* with a total of three. However, the majority of the chromosomes in the three species contained only one PYL gene, and multiple chromosomes were found to have no PYL gene (Figure S1).

### 3.2. A Molecular Characterization Analysis of PYLs in Three Ipomoea Species

Then, the sequence characteristics, including the length of amino acid residues, molecular weight, isoelectric point, and phosphorylation sites of PYL proteins in the three *Ipomoea* species, were compared. The length of amino acid sequences had obvious variations as the IbPYLs ranged from 123 to 244 aa, with an average of 204 aa, while the ItfPYLs ranged from 176 to 589 aa, with an average of 230 aa, and the ItbPYLs ranged from 185 to 231 aa, with an average of 205 aa, respectively. Accordingly, the molecular weight ranged from 13,787.9 to 28,064.36 Da, 19,641.92 to 66,277.93 Da, and 20,820.62 to 25,797.45 Da for *I. batatas*, *I. trifida*, and *I. triloba*, respectively. Additionally, the prediction of potential phosphorylation sites showed that the IbPYLs, ItfPYLs, and ItbPYLs contained 9 to 37, 14 to 65, and 14 to 30 possible phosphorylation sites, respectively (Table 1).

### 3.3. A Phylogenetic Analysis of PYL Proteins in Three Ipomoea Species

**Figure 1:**
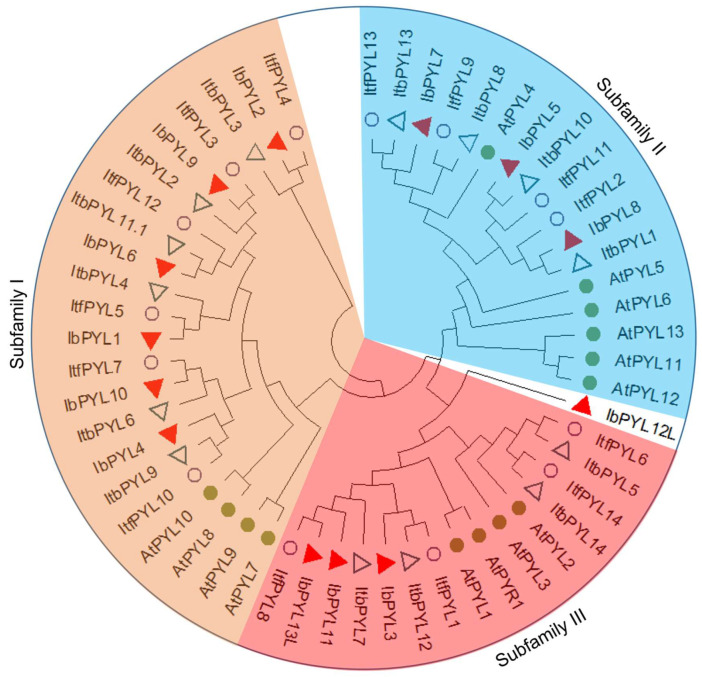
Phylogenetic analysis of PYL gene family in sweetpotato, its two diploid wild relatives, and Arabidopsis. The phylogenetic tree was conducted using MEGA X through the maximum likelihood method and the best evolutionary model WAG + G was employed with 1000 bootstraps. Different subgroups (I, II, and III) are named based on the reports in Arabidopsis, and are distinguished with different colors. Red triangles, hollow circles, hollow triangles, and green circles represent PYL protein sequences from *I. batatas*, *I. trifida*, *I. triloba*, and Arabidopsis, respectively.

The AtPYLs of Arabidopsis have been classified into three subfamilies based on their sequence similarity [9]. To explore the phylogenetic relatedness of PYL genes in the three *Ipomoea* species, a maximum likelihood method tree was constructed with their complete protein sequences of the identified 13 IbPYLs, 14 ItfPYLs, and 14 ItbPYLs, combined with 14 AtPYLs. Consistent with the results in Arabidopsis, these PYLs were also classified into three main subfamilies, except IbPYL12L did not belong to any of the subgroups due to its unique sequence composition. Generally, the distributions of PYLs in different subgroups was relatively uniform. Among them, subfamily I contained 18 *Ipomoea* proteins (six IbPYLs, six ItfPYLs, and six ItbPYLs), subfamily II included 11 *Ipomoea* proteins (three IbPYLs, four ItfPYLs, and four ItbPYLs), and the remaining 46 *Ipomoea* proteins belonged to subfamily III (three IbPYLs, four ItfPYLs and four ItbPYLs), respectively (Figure 1).

### 3.4. Gene Duplication and Collinearity Survey of PYL Genes

**Figure 2:**
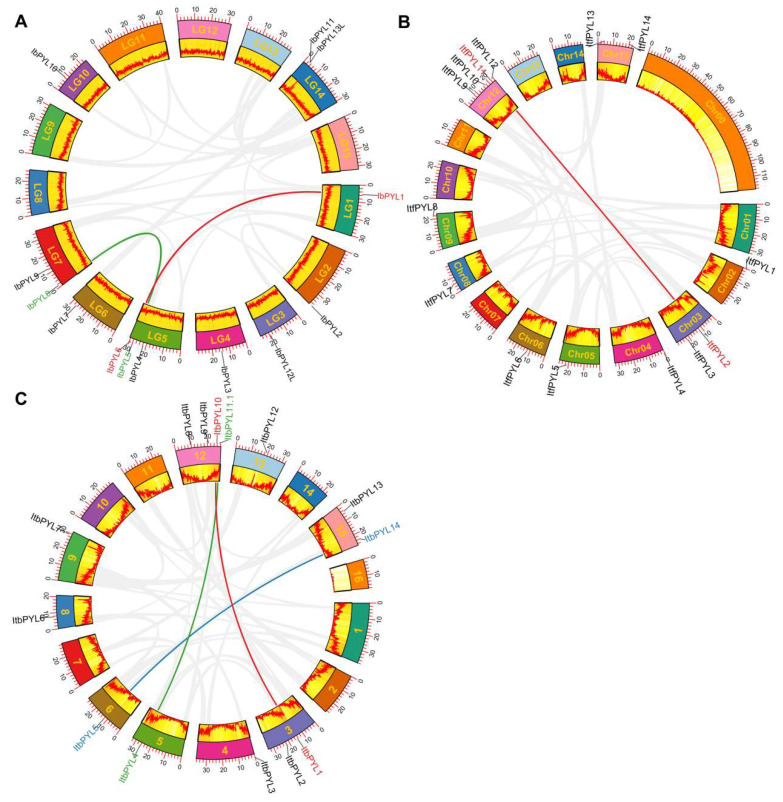
Circular visualizations of PYL genes in *I. batatas* (**A**), *I. trifida* (**B**), and *I. triloba* (**C**). Duplicated PYL gene pairs are represented by colored lines, and these genes are also marked with different colors.

The gene duplication events were detected to explore the possible gene expansion mechanism of PYLs in the three *Ipomoea* species. No tandem duplication events were found among the 41 PYLs, and a total of six gene pairs were recognized as segmental duplications among the 41 PYLs from the three *Ipomoea* species as follows: *IbPYL1*-*IbPYL6* and *IbPYL5*-*IbPYL8* in *I. batatas*; *ItfPYL2*-*ItfPYL11* in *I. trifid;* and *ItbPYL1*-*ItbPYL10*, *ItbPYL4*-*ItbPYL11.1,* and *ItbPYL5*-*ItbPYL14* in *I. triloba* (Figure 2).

**Figure 3:**
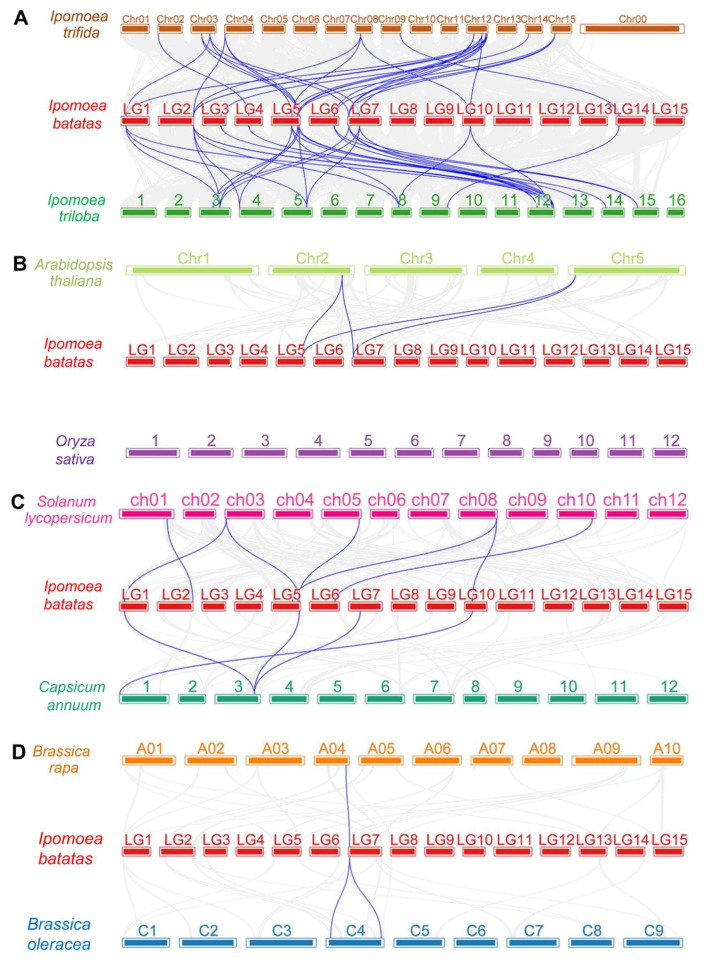
Syntenic relationships of PYL genes between sweetpotato and eight representative plants including *Ipomoea trifida* and *Ipomoea triloba* (**A**), *Arabidopsis thaliana* and *Oryza sativa* (**B**), *Solanum lycopersicum* and *Capsicum annuum* (**C**), and *Brassica rapa* and *Brassica oleracea* (**D**). The blue lines connecting two different chromosomes represent the syntenic PYL gene pairs within sweetpotato and other plant genomes.

Furthermore, to assess the potential evolutionary relationships of sweetpotato *IbPYL* genes, a collinearity survey between *IbPYLs* and orthologous genes from eight plants including *I. trifida*, *I. triloba*, *Arabidopsis thaliana, Oryza sayiva*, *Solanum lycopersicum*, *Capsicum annuum*, *Brassica rapa*, and *Brassica oleracea* was conducted. The results showed that there were 29 and 32 homologous gene pairs between sweetpotato and *I. trifida* and *I. triloba*, respectively, because most of the genes from *I. trifida* (12/14) and *I. triloba* (13/14) are collinear with two to three *IbPYLs* in sweetpotato. However, only four, seven, four, one, and two syntenic relationships were found between sweetpotato and *Arabidopsis thaliana*, *Solanum lycopersicum*, *Capsicum annuum*, *Brassica rapa*, and *Brassica oleracea*, respectively, and no such homologous gene pair was detected between sweetpotato and rice (Figure 3). The results show that among eight plant species examined, *I. batatas* has the largest number of homologs to *I. trifida* and *I. triloba*, supporting the fact that they are indeed the likely diploid wild relative of sweetpotato.

### 3.5. Conserved Domain and Gene Organization Analyses of PYL Proteins in Three Ipomoea Species

**Figure 4:**
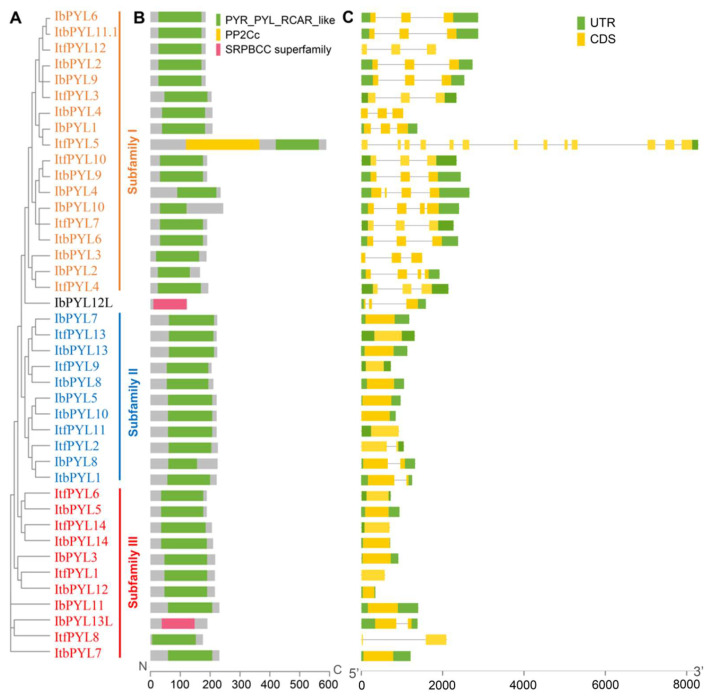
Phylogenetic relationship (**A**), conserved domain (**B**), and gene architecture (**C**) of PYL genes in sweetpotato and its two diploid wild relatives. The phylogenetic tree was constructed with MEGA X based on the same parameters used in Figure 1. The conserved domain analysis of the PYLs was identified using NCBI Batch CD-Search. Boxes of different colors present different domains, and the green box represents the PYL domain. The green and yellow rectangles represent the CDS and UTR, respectively.

To better visualize the gene organization and conserved domain, a phylogenetic tree was also generated using the complete protein sequences of the identified 13 IbPYLs, 14 ItfPYLs, and 14 ItbPYLs (Figure 4A). The results detected using NCBI CD-Search display that all the identified PYL proteins from the three *Ipomoea* species contained one conserved PYR_PYL_RCAR_like domain that characterizes the PYL protein except the IbPYL12L, and IbPYL13L contained the SRPBCC superfamily domain. Moreover, ItfPYL5 from *I. trifida* contained an additional PP2C conserved domain (Figure 4B).

The exon–intron structures are significant components of genes and also provide important clues for functional diversifications of genes. Thus, the gene structures of these PYL genes were further analyzed. It is obvious that the PYL genes from the same subfamily revealed similar gene organizations. Among them, the PYL genes from subfamilies II and III were both intronless, of which only five PYL members had only one intron. Contrarily, most PYL members in subfamily I had two introns, except ItfPYL5, IbPYL2, IbPYL4, and IbPYL10. Moreover, the gene length and gene structure of most sweetpotato *IbPYLs* were similar to their orthologies in diploid wild relative *I. trifida* and *I. triloba* (Figure 4C).

### 3.6. Conserved Motif Analyses of PYL Proteins in Three Ipomoea Species

**Figure 5:**
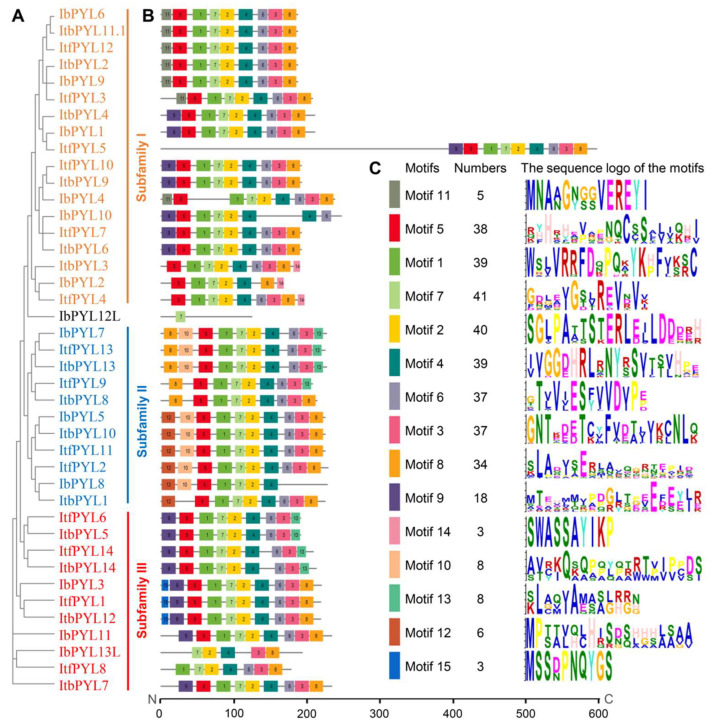
Phylogenetic relationship (**A**), conserved motif (**B**), and sequence logo of conserved motif (**C**) of PYL proteins in sweetpotato and its two diploid wild relatives. The phylogenetic tree was constructed with MEGA X based on the same parameters used in Figure 1. Conserved motif compositions within PYL proteins were identified using MEME; the different colors represent the 15 identified motifs. The sequence logo of conserved motifs represents the amino acid conservation.

To further compare the sequence composition of the PYL proteins in three *Ipomoea* species, their conserved motifs were predicted with the MEME tool using the parameter reported in rice [12], and a total of 15 conserved motifs were identified. Similar to the gene structure, generally, the PYL members showing a closer phylogenetic relationship had more similarly conserved motif compositions, which further supports the phylogenetic analysis results (Figure 5). The results show that most PYL members had nine conserved motifs, but they displayed slight differences in their specific motif compositions. For instance, IbPYL13L and IbPYL10 had five and eight motifs, respectively, while IbPYL12L contained only one motif. Multiple motifs existed, specifically in several members of the different subgroups, such as motif 12 and motif 15. The data suggest that the structural complexity of PYL genes and the specific motifs in different subgroups might play important roles in their evolutions and functions.

### 3.7. The Expression Profiles of IbPYLs under Abiotic Stress

**Figure 6:**
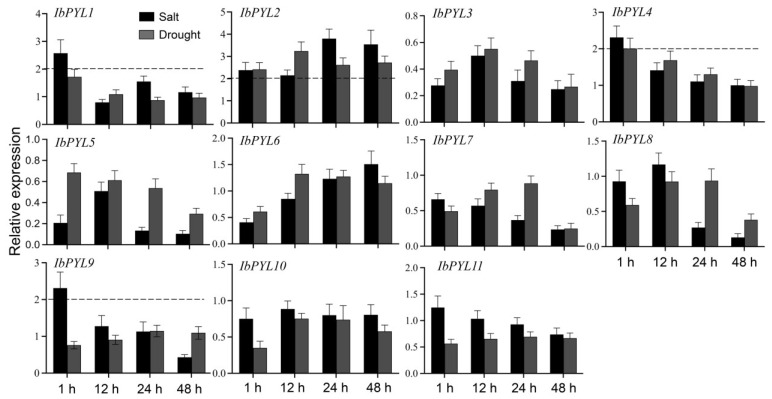
The expression of *IbPYL* genes in response to salt and dehydration treatments found using qRT-PCR analysis. The *Y*-axis delineates the fold changes in relative expression compared with 0 h, and the data are normalized to 1. Bars represent the mean of three biological replicates ± SE. The two-fold threshold is presented by a dotted line.

qRT-PCR assays were conducted to detect the expression profiles of 11 *IbPYLs* except *IbPYL12L* and *IbPYL13L* under abiotic stresses including NaCl and PEG6000 (dehydration stress). Considering the biological significance, a two-fold cut-off value was adopted. As depicted in Figure 6, the transcription level of *IbPYL2* and *IbPYL4* could be induced by both NaCl and PEG6000 stresses, and the upregulated expression of *IbPYL1* and *IbPYL9* could also be detected under salt stress. No obvious upregulation was observed in the expression of other *IbPYLs* since the induction level was less than two-fold. These results suggest that sweetpotato *IbPYL* genes may participate in the response to abiotic stress.

## 4. Discussion

ABA is one of the most critical regulators to modulate key and diverse processes during plant growth and development and stress response [5,6]. ABA is perceived by the PYL receptor, which is the biggest plant hormone receptor family and the crucial component of ABA signal transduction pathway [34]. Arduous efforts have been made to characterize the functions of PYL genes, mainly in Arabidopsis, while only very limited progress has been made concerning the study of PYL genes in most crops. *Ipomoea* is the most prevalent species in the Convolvulaceae family, which is widely used and of great value in industry and agriculture [19,21], while, to date, a genome-wide analysis of PYL genes in *Ipomoea* has not been reported.

Recently, different amounts of the PYL genes have been identified in many plant species, including 10 in *Glycyrrhiza uralensis* [35], 13 in rice [12], 13 in apple [36], 14 in *Arabidopsis* [9], 14 in tea [37], 14 in cucumber [14], and 46 in *Brassica napus* [11]. In this study, a genome-wide comprehensive analysis of PYL genes in cultivated sweetpotato and its two diploid wild relatives, *I. trifida* and *I. triloba*, about their potential roles in stress response was carried out via bioinformatics and experimental methods. Similar numbers of PYL genes were identified in the genomes of sweetpotato, *I. trifida*, and *I. triloba*, namely 13, 14, and 14, respectively. However, previous results showed that polyploidization plays important roles in the expansion of PYL genes. For instance, 29 PYLs were identified in the genomes of allotetraploid tobacco, while only 11 or 16 PYLs were identified in its two diploid ancestors [11]. Similarly, 39 or 40 PYLs were identified in tetraploid cotton, whereas only 20 or 21 were identified in its diploid progenitors [13], and 38 PYLs were isolated in allohexaploid wheat as opposed to only 12 or 26 in its progenitors [15]. The absence of significant PYL expansion in cultivated sweetpotato compared with diploid species may be due to the limitations of the half haplotype-resolved hexaploid genome of sweetpotato [22,38].

A total of 41 PYLs from the three *Ipomoea* species were classified into three subfamilies: I, II, and III based on the phylogenetic relationship reported in Arabidopsis [9]. We found that the identified *Ipomoea* PYLs have specific subfamily features and the PYLs classified into the same subfamily generally share similar protein motifs. For instance, the *Ipomoea* PYLs from subfamily II and III are intronless, while all members belonging to subfamily I have multiple introns. The gene structures in the surveyed *Ipomoea* PYLs were similar to those orthologs in Arabidopsis, rice, apple, and *Medicago sativa* [12,36,39]. The results suggest that the gene organizations of *Ipomoea* PYLs are closely associated with the phylogenetic relationship of the genes. Moreover, genome duplications play key roles in promoting the evolution and expansion of gene families [40]. In this study, fragment duplication was identified as having a prominent role in *Ipomoea* PYL gene expansion. Similar situations were also reported in the PYLs from apple, cucumber, *Medicago sativa,* and *Prunus avium* [14,36,39,41]. Moreover, there is the largest number of collinear gene pairs between *I. batatas* and *I. trifida, I. triloba*, indicating that their two diploid wild relatives play key roles in the evolution of sweetpotato PYL genes, which is consistent with that of our previous reports of multiple transcription factor families [28,38,42].

ABA is a vital well-established stress hormone that plays a key role in plant response to abiotic stresses [43,44]. And the role of ABA receptor PYLs in abiotic stress tolerance has also been widely verified in many plants. For example, transgenic plants overexpressing Arabidopsis *PYL13* showed enhanced ABA sensitivity, increased water use efficiency, enhanced drought tolerance, and accelerated stress-related gene expression, and *AtPYL9* can also provide drought tolerance [16,17]. Rice *OsPYL5* and *OsPYL10* and cotton *PYL26* were functionally characterized to affect abiotic stress tolerance including drought and/or cold [45,46,47]. Recently, *MdPYL9* was determined to improve the drought tolerance of transgenic apple plants [48]. In this study, the transcription profiles of *IbPYLs* under abiotic stress treatments were analyzed using qRT-PCR, and we found that the expression of multiple *IbPYLs* was induced by drought and/or salt stresses, implying their possible involvement in abiotic stress responses. Interestingly, the transcription levels of several *IbPYL* members were downregulated by abiotic stress. Similarly, the transcription of PYLs in Arabidopsis was also reduced by stress, while the overexpression of *AtPYL5* or *AtPYL9* still improved drought tolerance [16,49,50]. In our study, phylogenetic analysis showed that the IbPYL5, IbPYL7, and IbPYL8 proteins were closed linked to AtPYL4 and AtPYL5, which were monomeric-type ABA receptors [51]. It is worth mentioning that the overexpression of tomato monomeric-type, rather than dimeric ABA receptors in Arabidopsis, could enhance drought stress tolerance [52]. Therefore, the downregulated *IbPYLs* may also be important candidate ABA receptors in response to stress, while their specific functions in stress tolerance require further experimental validation.

## 5. Conclusions

This study systematically characterized the genome organization, gene structure, conserved domain, molecular evolution, and expression profiles of ABA receptor PYL genes in *Ipomoea* species. The identified 41 PYL proteins were classified into three subfamilies that are structurally conserved, and segmental duplication was identified as the dominant driving force for the expansion of PYLs. Multiple stress-induced *IbPYLs* might be related to abiotic stress responses in sweetpotato. These findings provide valuable information on the evolutionary relationship of PYL genes in *Ipomoea* species, and further validation of candidate *IbPYL* genes in stress tolerance is necessary.

## Figures and Tables

**Table 1:** Characteristics of PYL proteins in three *Ipomoea* species.

Gene Name	Gene ID	Amino Acids	MW (Da)	PI	No. of Phosphorylation Site			
					Ser Site	Tyr Site	Thr Site	Total
*Ipomoea batatas*								
*IbPYL1*	g928.t1	208	23,607.73	6.17	9	3	6	18
*IbPYL2*	g9406.t1	166	18,635.23	5.5	7	3	5	15
*IbPYL3*	g14758.t1	217	24,651.82	9.88	17	1	11	29
*IbPYL4*	g19566.t1	235	26,974.6	5.36	9	3	5	17
*IbPYL5*	g20407.t1	222	24,209.3	6.72	13	0	8	21
*IbPYL6*	g20643.t1	185	20,584.35	5.8	12	2	4	18
*IbPYL7*	g24506.t1	224	24,246.15	5.5	14	1	11	26
*IbPYL8*	g25480.t1	225	24,211.46	9.01	22	3	12	37
*IbPYL9*	g26930.t1	185	20,834.65	6.17	12	3	4	19
*IbPYL10*	g39321.t1	244	28,064.36	6.85	9	3	4	16
*IbPYL11*	g55788.t1	231	25,788.44	5.38	15	1	14	30
*IbPYL12L*	g12068.t1	123	13,787.9	5.72	4	1	4	9
*IbPYL13L*	g55782.t1	191	21,202.85	8.74	8	1	9	18
*Ipomoea trifida*								
*ItfPYL1*	itf02g00240.t1	216	23,545.22	6.19	15	1	11	27
*ItfPYL2*	itf03g14430.t1	226	24,533.66	6.58	10	0	8	18
*ItfPYL3*	itf03g25530.t1	205	23,146.29	6.39	12	3	5	20
*ItfPYL4*	itf04g00430.t1	194	21,674.61	5.23	7	4	7	18
*ItfPYL5*	itf05g19440.t1	589	66,277.93	5.96	36	9	20	65
*ItfPYL6*	itf06g09740.t1	189	21,208.09	5.86	13	1	9	23
*ItfPYL7*	itf08g08150.t1	190	21,766.77	6.07	8	2	4	14
*ItfPYL8*	itf09g25130.t1	176	19,641.92	6.07	8	1	12	21
*ItfPYL9*	itf12g11260.t1	205	22,234.04	8.26	18	0	10	28
*ItfPYL10*	itf12g17820.t1	190	21,696.61	6.3	9	2	4	15
*ItfPYL11*	itf12g23670.t1	222	24,274.38	6.55	12	0	7	19
*ItfPYL12*	itf12g25980.t1	185	20,568.35	5.8	10	2	4	16
*ItfPYL13*	itf15g06620.t1	222	23,971.92	5.8	13	1	11	25
*ItfPYL14*	itf15g20740.t1	206	22,708.42	6.35	9	2	9	20
*Ipomoea triloba*								
*ItbPYL1*	itb03g15060.t1	222	24,141.25	6.62	9	0	7	16
*ItbPYL2*	itb03g23630.t1	185	20,820.62	6.16	12	3	4	19
*ItbPYL3*	itb04g00250.t1	188	20,930.69	5.34	7	4	7	18
*ItbPYL4*	itb05g20040.t1	208	23,575.67	6.17	9	3	6	18
*ItbPYL5*	itb06g07990.t1	189	21,208.09	5.86	13	1	9	23
*ItbPYL6*	itb08g08610.t1	190	21,766.77	6.07	8	2	4	14
*ItbPYL7*	itb09g28750.t1	231	25,797.45	5.46	15	1	14	30
*ItbPYL8*	itb12g11410.t1	211	22,806.61	6.15	18	0	12	30
*ItbPYL9*	itb12g18500.t1	190	21,696.61	6.3	9	2	4	15
*ItbPYL10*	itb12g24010.t1	222	24,176.31	6.72	14	0	7	21
*ItbPYL11.1*	itb12g26370.t1	185	20,616.41	5.8	12	2	4	18
*ItbPYL11.2*	itb12g26370.t2	185	20,616.41	5.8	12	2	4	18
*ItbPYL12*	itb13g12810.t1	216	23,516.19	6.36	15	0	11	26
*ItbPYL13*	itb15g06930.t1	224	24,172.11	5.64	13	1	11	25
*ItbPYL14*	itb15g21060.t1	210	23,078.74	6.55	9	2	9	20

## Data Availability

The data that supports the findings of this study are available from the corresponding authors.

## Supplementary Materials

The following supporting information can be downloaded at: https://www.mdpi.com/article/10.3390/genes14071471/s1. Figure S1: Distribution of the identified 13 *IbPYLs* in *I. batatas*, 14 *ItfPYLs* in *I. trifida*, and 14 *ItbPYLs* in *I. triloba* across their genomes. Table S1: The amino acid sequences of reported PYLs identified in the genomes of six plant species including Arabidopsis, rice, wheat, maize, barley, and sorghum. Table S2: The nucleotide and amino acid sequences of PYLs identified in the genomes of three *Ipomoea* species. Table S3: Specific primer sequences used for qRT-PCR analysis.
