# Supplementary material for: Comparative Analysis of the PYL Gene Family in Three Ipomoea Species and the Expression Profiling of IbPYL Genes during Abiotic Stress Response in Sweetpotato
Source: Genes (Basel). 2023 Jul 19;14(7):1471. doi: 10.3390/genes14071471 (PMC10379866; doi:10.3390/genes14071471)
Supplement: Supplementary file 1 [file genes-14-01471-s001.zip › genes-2499347-supplementary.pdf]

Figure S1. Distribution of the identified 13 *IbPYLs* in *Ipomoea batatas* (A), 14 *ItfPYLs* in *Ipomoea trifida* (B), 14 *ItbPYLs* in *Ipomoea triloba* (C) across their genomes. The chromosome numbers are indicated to the left of each chromosome, and all chromosomes are drawn to scale based on their actual physical lengths.

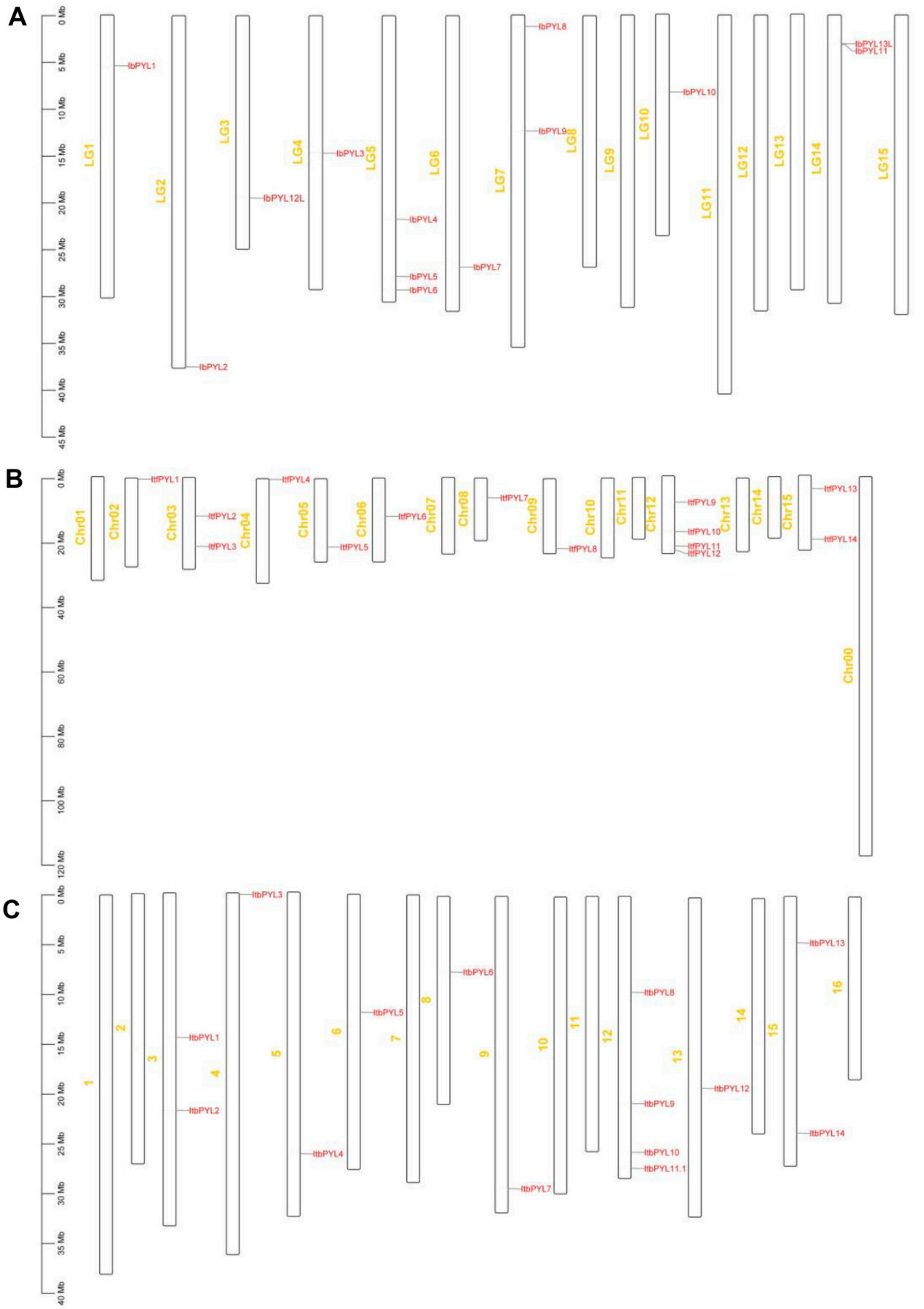

Table S1. The amino acid sequences of reported PYLs identified in the genomes of six plant species including *Arabidopsis*, rice, wheat, maize, barley and sorghum.

>AtPYR1
MPSELTPEERSELKNSIAEFHTYQLDPGSCSSLHAQRIHAPPELVWSIVRRFDKPQTYKHFIKSCS
VEQNFEMRVGCTRDVIVISGLPANTSTERLDILDDERRVTGFSIIGGEHRLTNYKSVTTVHRFEK
ENRIWTVVLESYVVDMPEGNSEDDTRMFADTVVKLNLQKLATVAEAMARNSGDGSGSQVT
>AtPYL1
MANSESSSSPVNEEENSQRISTLHHQTMPSDLTQDEFTQLSQSIAEFHTYQLGNGRCSSLLAQRI
HAPPETVWSVVRRFDRPQIYKHFIKSCNVSEDFEMRVGCTRDVNVISGLPANTSRERLDLLDD
DRRVTGFSITGGEHRLRNYKSVTTVHRFEKEEEEERIWTVVLESYVVDVPEGNSEEDTRLFADT
VIRLNLQKLASITEAMNRNNNNNNNSSQVR
>AtPYL2
MSSSPAVKGLTDEEQKTLEPVIKTYHQFEPDPTTCTSLITQRIHAPASVVWPLIRRFDNPERYKHF
VKRCRLISGDGDVGSVREVTVISGLPASTSTERLEFVDDDHRVLSFRVVGGEHRLKNYKSVTSV
NEFLNQDSGKVYTVVLESYTVDIPEGNTEEDTKMFVDTVVKLNLQKLGVAATSAPMHDDE
>AtPYL3
MNLAPIHDPSSSSTTTTSSSTPYGLTKDEFSTLDSIIRTHHTFPRSPNTCTSLIAHRVDAPAHAIWR
FVRDFANPNKYKHFIKSCTIRVNGNGIKEIKVGTIREVSVVSGLPASTSVEILEVLDEEKRILSFR
VLGGEHRLNNYRSVTSVNEFVVLEKDKKKRVYSVVLESYIVDIPQGNTEEDTRMFVDTVVKS
NLQNLAVISTASPT
>AtPYL4
MLAVHRPSSAVSDGDSVQIPMMIASFQKRFPSLSRDSTAARFHTHEVGPNQCCSAVIQEISAPIST
VWSVVRRFDNPQAYKHFLKSCSVIGGDGDNVGSLRQVHVVSGLPAASSTERLDILDDERHVIS
FSVVGGDHRLSNYRSVTTLHPSPISGTVVVESYVVDVPPGNTKEETCDFVDVIVRCNLQSLAKI
AENTAAESKKKMSL
>AtPYL5
MRSPVQLQHGSDATNGFHTLQPHDQTDGPIKRVCLTRGMHVPEHVAMHHTHDVGPDQCCSSV
VQMIHAPPESVWALVRRFDNPKVYKNFIRQCRIVQGDGLHVGDLREVMVVSGLPAVSSTERLE
ILDEERHVISFSVVGGDHRLKNYRSVTTLHASDDEGTVVVESYIVDVPPGNTEEETLSFVDTIV
RCNLQSLARSTNRQ
>AtPYL6
MPTSIQFQRSSTAAEAANATVRNYPHHHQKQVQKVSLTRGMADVPEHVELSHTHVVGPSQCF
SVVVQDVEAPVSTVWSILSRFEHPQAYKHFVKSCHVVIGDGREVGSVREVRVVSGLPAAFSLE
RLEIMDDDRHVISFSVVGGDHRLMNYKSVTTVHESEEDSDGKKRTRVVESYVVDVPAGNDKE
ETCSFADTIVRCNLQSLAKLAENTSKFS
>AtPYL7
MEMIGGDDTDTEMYGALVTAQSLRLRHLHHCRENQCTSVLVKYIQAPVHLVWSLVRRFDQPQ
KYKPFISRCTVNGDPEIGCLREVNVKSGLPATTSTERLEQLDDEEHILGINIIGGDHRLKNYSSIL
TVHPEMIDGRSGTMVMESFVVDVPQGNTKDDTCYFVESLIKCNLKSLACVSERLAAQDITNSI
ATFCNASNGYREKNHTETNL
>AtPYL8
MEANGIENLTNPNQEREFIRRHHKHELVDNQCSSTLVKHINAPVHIVWSLVRRFDQPQKYKPFI
SRCVVKGNMEIGTVREVDVKSGLPATRSTERLELLDDNEHILSIRIVGGDHRLKNYSSIISLHPET
IEGRIGTLVIESFVVDVPEGNTKDETCYFVEALIKCNLKSLADISERLAVQDTTESRV

>AtPYL9
MMDGVEGGTAMYGGLETVQYVRTHHQHLCRENQCTSALVKHIKAPLHLVWSLVRRFDQPQK
YKPFVSRCTVIGDPEIGSLREVNVKSGLPATTSTERLELLDDEEHILGIKIIGGDHRLKNYSSILTV
HPEIIEGRAGTMVIESFVVDVPQGNTKDETCYFVEALIRCNLKSLADVSERLASQDITQ
>AtPYL10
MNGDETKKVESEYIKKHHRHELVESQCSSTLVKHIKAPLHLVWSIVRRFDEPQKYKPFISRCVV
QGKKLEVGSVREVDLKSGLPATKSTEVLEILDDNEHILGIRIVGGDHRLKNYSSTISLHSETIDG
KTGTLAIESFVVDVPEGNTKEETCFFVEALIQCNLNSLADVTERLQAESMEKKI
>AtPYL11
METSQKYHTCGSTLVQTIDAPLSLVWSILRRFDNPQAYKQFVKTCNLSSGDGGEGSVREVTVV
SGLPAEFSRERLDELDDESHVMMISIIGGDHRLVNYRSKTMAFVAADTEEKTVVVESYVVDVP
EGNSEEETTSFADTIVGFNLKSLAKLSERVAHLKL
>AtPYL12
MKTSQEQHVCGSTVVQTINAPLPLVWSILRRFDNPKTFKHFVKTCKLRSGDGGEGSVREVTVV
SDLPASFSLERLDELDDESHVMVISIIGGDHRLVNYQSKTTVFVAAEEEKTVVVESYVVDVPEG
NTEEETTLFADTIVGCNLRSLAKLSEKMMELT
>AtPYL13
MESSKQKRCRSSVVETIEAPLPLVWSILRSFDKPQAYQRFVKSCTMRSGGGGGKGGEGKGSVR
DVTLVSGFPADFSTERLEELDDESHVMVVSIIGGNHRLVNYKSKTKVVASPEDMAKKTVVVES
YVVDVPEGTSEEDTIFFVDNIIRYNLTSLAKLTKKMMK

>OsPYL1
MEQQEEVPPPPAGLGLTAEEYAQVRATVEAHHRYAVGPGQCSSLLAQRIHAPPAAVWAVVRRF
DCPQVYKHFIRSCVLRPDPHHDDNGNDLRPGRLREVSVISGLPASTSTERLDLLDDAHRVFGFT
ITGGEHRLRNYRSVTTVSQLDEICTLVLESYIVDVPDGNTEDDTRLFADTVIRLNLQKLKSVSEA
NANAAAAAAAPPPPPPAAAE
>OsPYL2
MEAHVERALREGLTEEERAALEPAVMAHHTFPPSTTTATTAAATCTSLVTQRVAAPVRAVWPIV
RSFGNPQRYKHFVRTCALAAGDGASVGSVREVTVVSGLPASTSTERLEMLDDDRHIISFRVVG
GQHRLRNYRSVTSVTEFQPPAAGPAPAPPYCVVVESYVVDVPDGNTAEDTRMFTDTVVKLNL
QKLAAVAEDSSSASRRRD
>OsPYL3
MEPHMERALREAVASEAERRELEGVVRAHHTFPAAERAAGPGRRPTCTSLVAQRVDAPLAAV
WPIVRGFANPQRYKHFIKSCELAAGDGATVGSVREAVVSGLPASTSTERLEILDDDRHVLSFR
VVGGDHRLRNYRSVTSVTEFSSPSSPPSPPRPYCVVVESYVVDVPEGNTEEDTRMFTDTVVKL
NLQKLAAVATSSSPPAAGNHH
>OsPYL4
MPYAAVRPSPPPQLSRPIGSGAGGGKACPAVPCEVARYHEHAVGAGQCCSTVVQAIAAPADAV
WSVVRRFDRPQAYKKFIKSCRLVDGDGGEVGSVREVRVVSGLPATSSRERLEVLDDDRRVLSF
RIVGGEHRLANYRSVTTVHEAAAPAMAVVVESYVVDVPPGNTWEETRVFVDTIVRCNLQSLA
RTVERLAPEAPRANGSIDHA
>OsPYL5
MMPYTAPRPSPPQHSRIGGCGGGGVLKAAGAAGHAASCVAVPAEVARHHEHAAGVGQCCSA
VVQAIAAPVDAVWSVVRRFDRPQAYKHFIRSCRLLDGDGDGGAVAVGSVREVRVVSGLPATSS

RERLEILDDERRVLSFRVVGGEHRLSNYRSVTTVHETAAGAAAAVVVESYVVDVPHGNTADET
RMFVDTIVRCNLQSLARTAEQLALAAPRAA
>OsPYL6
MPCIPASSPGIPHQHQHQHHRALAGVGMAVGCAAEAAVAAAGVAGTRCGAHDGEVPMEVAR
HHEHAEPGSGRCCSAVVQHVAAPAPAVWSVVRRFDQPQAYKRFVRSCALLAGDGGVGTLREV
RVVSGLPAASSRERLEILDDESHVLSFRVVGGEHRLKNYLSVTTVHPSPSAPTAATVVVESYVV
DVPPGNTPEDTRVFVDTIVKCNLQSLANTAEKLAAGARAAGS
>OsPYL7
MNSGAGGAGGAAVGRMPAGSLQWAQWRLADERCELREEEMEYMRRFHRHEIGSNQCNSFIA
KHVRAPLQNVWSLVRRFDQPQIYKPFVRKCVMRGNVETGSVREIIVQSGLPATRSIERLEFLDD
NEYILRVKFIGGDHMLKKCGP
>OsPYL8
MNGAGGAGGAAAGKLPMVSHRQVQWRLADERCELREEEMEYIRQFHRHEPSSNQCTSFVAK
HIKAPLQTVWSLVRRFDQPQLFKPFVRKCVMRENIIATGCVREVNVQSGLPATRSTERLELLDD
NEHILKVKFIGGDHMLKNYSSILTIHSEVIDGQLGTLVVESFVVDIPEGNTKDDICYFIENILRCN
LMTLADVSEERLANP
>OsPYL9
MNGVGGAGGAAAGKLPMVSHRRVQWRLADERCELREEEMEYIRRFHRHEPSSNQCTSFAAK
HIKAPLHTVWSLVRRFDQPQLFKPFVRNCVMRENIIATGCIREVNVQSGLPATRSTERLELLDD
NEHILKVKFIGGDHMLKNYSSILTIHSEVIDGQLGTLVVESFIVDVLEGNTKDDISYFIENVLRCN
LRTLADVSEERLANP
>OsPYL10
MVEVGGGAAEAAAGRRWRLADERCDLRAAETEYVRRFHRHEPRDHQCSSAVAKHIKAPVHL
VWSLVRRFDQPQLFKPFVSRCEMKGNIEIGSVREVNVKSGLPATRSTERLELLDDNEHILSVRF
VGGDHRLKNYSSILTVHPEVIDGRPGTLVIESFVVDVPEGNTKDETCYFVEALLKCNLKSLAEV
SERLVVKDQTEPLDR
>OsPYL11
MVGLVGGGGWRVGDDAAGGGGGGAVAAGAAAAAEAEHMRRLHSHAPGEHQCSSALVKHIK
APVHLVWSLVRSFDQPQRYKPFVSRCVVRGGDLEIGSVREVNVKTGLPATTSTERLELLDDDE
HILSVKFVGGDHRLRNYSSIITVHPESIDGRPGTLVIESFVVDVPDGNTKDETCYFVEAVIKCNLT
SLAEVSERLAVQSPTSPLEQ
>OsPYL12
MRGSTSLAVGCVREVDFKSGFPAKSSVERLEILDDKEHVFGVRIIGGDHRLKNYSSVLTAKPEVI
DGEPATLVSESFVVDVPEGNTADETRHFVEFLIRCNLRSLAMVSQRLLLAQGDLAEPPAQ
>OsPYL13
MNGCTGGAGGVAAGRLPAVSLQQAQWKLVDERCELREEEMEYVRWFHRYELVATGATPSLPN
TSGCPSKLGLPSTRRIERLGFPDDNDHTLRVKFIGGDHMLKDYSSTLIIHLEVIDGQLVTLVIESF
VVDILEGNTKDEISYFIENLLKFNLRTLRV

>TaPYL1A
MEQQPVAAAEPEVPAGLGLTAAEYAQLLPTVEAYHRYAVGPGQCSSLVAQRIEAPPAAVWAIVR
RFDCPQVYKHFIRSCALRPDPEAGDELRPGRLREVSVISGLPASTSTERLDLLDDARRAFGFTIT
GGEHRLRNYRSVTTVSELSPAAPAEICTVVLESYVVDVPDGNSEEDTRLFADTVVRLNLQKLK

SVAEANAAAAAATPAPPAE
>TaPYL1B
MEQQPVAAAAAAEPEVPAGLGLTAAEYAQLLPTVEAYHRYAVGPGQCSSLVAQRIEAPPAAVW
AIVRRFDCPQVYKHFIRSCALRPDPEAGDELRPGRLREVSVISGLPASTSTERLDLLDDARRAFG
FTITGGEHRLRNYRSVTTVSELSPAAPAEICTVVLESYVVDVPDGNSEEDTRLFADTVVRLNLQ
KLKSVAEANAAAAATTAPPAE
>TaPYL1D
MEQQPVAAAATEPEVPAGLGLTAAEYAQLLPTVEAYHRYAVGPGQCSSLVAQRIEAPPAAVWAI
VRRFDCPQVYKHFIRSCALRPDPEAGDELRPGRLREVSVISGLPASTSTERLDLLDDARRAFGFT
ITGGEHRLRNYRSVTTVSELSPAAPAEICTVVLESYVVDVPDGNSEEDTRLFADTVVRLNLQKL
KSVAEANAAAAAATPAPPAE
>TaPYL2A
MESALRQGLTEPERREVEGVVEEHHTFPGRASGTCTSLVTQRVQAPLAAVWDIVRGFANPQRY
KHFIKSCALAAGDGATVGSVREVTVVSGLPASTSTERLEILDDDRHILSFRVVGGEHRLRNYRS
VTSVTEFTDQPSGPPYCVVVESYVVDVPEGNTEEDTRMFTDTVVKLNLQKLAAIATTTSSSSPP
PSDEQS
>TaPYL2D
MESALRQGLTEPERREVEGVVEEHHTFPGRASGTCTSLVTQRVQAPLAAVWDIVRGFANPQRY
KHFIKSCALAAGDGATVGSVREVTVVSGLPASTSTERLEILDDDRHILSFRVVGGEHRLRNYRS
VTSVTEFADEPSGPSYCVVVESYVVDVPEGNTEEDTRMFTDTVVKLNLQKLAAIATTTTSSSPP
PSDEQS
>TaPYL3A
MEAHMERALREGVTEAERAALEGTVRAHHTFPGRAPGGTCTSLVAQRVAAPVRAVWPIVRSF
GNPQRYKHFVRTCALAAGDGASVGSVREVTVVSGLPASTSTERLEILDDDRHILSFSVVGGDH
RLRNYRSVTSVTEFQPGPYCVVVESYVVDVPDGNTEEDTRMFTDTVVKLNLQKLASVAEDSA
AAPGSRRRD
>TaPYL3B
MMEAHMERALQEGVTEAERAALEGTVRAHHTFPGRVPGATCTSLVAQRVAAPVRAVWPIVRS
FGNPQRYKHFVRTCALAAGDGASVGSVREVTVVSGLPASTSTERLEILDDDRHILSFSVVGGEH
RLRNYRSVTSVTEFQPGPYCVVVESYVVDVPDGNTEEDTRMFTDTVVKLNLQKLASVAEETA
AAPGSRRRD
>TaPYL3D
MMEAHMEQALREGVTEAERAALEGTVRAHHTFPGRAPGATCTSLVAQRVAAPVRAVWPIVRS
FGNPQRYKHFVRTCALAAGDGASVGSVREVTVVSGLPASTSTERLEILDDDRHILSFSVVGGEH
RLRNYRSVTSVTEFQPGPYCVVVESYVVDVPEGNTEEDTRMFTDTVVKLNLQKLASVAEESA
AAPGSRRRD
>TaPYL4A
MPRRLAATGSALLPGEPQVLRTIYIVRLAPPTFNLLPNQAKRSSAAARSAPIPPNKQLAGEATRS
ALEPMPTPYSAAALQQHHRLVSSSGGLATAAAAGAHRCGEHDGTVPPEVARHHEHAAPGGRC
CCSAVVQRVAAPAADVWAVVRRFDQPQAYKSFVRSCALLDGDGGVGTLREVRVVSGLPAASS
RERLEILDDERHVLSFSVVGGEHRLRNYRSVTTVHPAPGESASATLVVESYVVDVPPGNTPEDT
RVFVDTIVKCNLQSLARTAEKLAGRGPAYGALP
>TaPYL4B
MPTPYSAAALQQHHRLVSSSGGLAVAAATGAHRCGEHDGTVPPEVARHHEHAAPGGRCCCSA

VVQRVAAPAADVWAVVRRFDQPQAYKSFVRSCALLDGDGGVGTLREVRVVSGLPAASSRERL
EILDDERHVLSFSVVGGEHRLRNYRSVTTVHPAPGGSASATLVVESYVVDVPPGNTPEDTRVFV
DTIVKCNLQSLARTAEKLAGRGAAYGALP
>TaPYL4D
MPTPYSAAALQQHQRLVSSSGGLAATGAHRCGEHDGTVPPEVARHHEHAAPGGRCCCSAVVQ
RVAAPAADVWAVVRRFDQPQAYKSFVRSCALLDGDGGVGTLREVRVVSGLPAASSRERLEILD
DERHVLSFSVVGGEHRLRNYRSVTTVHPAPGESASATLVVESYVVDVPPGNTPEDTRVFVDTIV
KCNLQSLARTAEKLAGRGAAYGALP
>TaPYL5A
MPYAAARPSPQQHSRISSGCKALVAHGAAVPGEVARYHEHAAGAGQCCSAVVQAIAAPVEAV
WSVVRRFDRPQAYKRFIKSCRMVDGDGGAVGSVREVRVVSGLPGTSSRERLEILDDERRVLSF
RIVGGEHRLANYRSVTTVSEVASTVAGAPRVTLVVESYVVDVPPGNTSDETRLFVDTIVRCNLQ
SLARTAEQLALAVPHVN
>TaPYL5B
MPYAAARPSLQQHSRISSGCKALVAHGAAVPGEVALYHEHAAGAGQCCSAVVQAIAAPVEAV
WSVVRRFDRPQAYKRFIKSCRVVDGDGGAVGSVREVRVVSGLPGTSSRERLEILDDERRVLSFR
IVGGEHRLANYRSVTTVNEVASTVAAGAPRVTLVVESYVVDVPPGNTSDETRLFVDTIVRCNL
QSLARTAEQLALAVPHVN
>TaPYL5D
MPYAAARPSPQQHSRISAGCKALVAHGAAVPGEVARYHEHAAGAGQCCSAVVQAIAAPVEAV
WSVVRRFDRPQAYKRFIKSCRLVDGDGGAVGSVREVRVVSGLPGTSSRERLEILDDERRVLSFR
IVGGEHRLANYRSVTTVSEVASTVAGAPRVTLVVESYVVDVPPGNTSDETRMFVDTIVRCNLQ
SLARTAEQLALAVPHVN
>TaPYL6A
MPYTASRPSAPQRARVAAGGGGWKAAAHAASCGAVPGEVARHHEHAAGAGQCCSAVVQAIE
APVGAVWAVVRRFDRPQAYKHFIRSCRVVDGDGGAVGSVREVRVVSGLPATSSRERLEILDDE
RRVLSFRVVGGEHRLSNYRSVTTVHEAASAGAVVVESYVVDVPPGNTADETRTFVDTIVRCNL
QSLARTAQQLALAA
>TaPYL6B
MPYTASRPSAPQRARVAAVGAGWKAAAHAASCGAVPGEVARHHEHAAGTGQCCSAVVQAIE
APVGAVWAVVRRFDRPQAYKHFIRSCRLVDGDGGAVGSVREVRVVSGLPATSSRERLEILDDER
RVLSFRVVGGEHRLSNYRSVTTVHEAASAGAVVVESYVVDVPPGNTADETRTFVDTIVRCNLQ
SLARTA
>TaPYL6D
MPYTASRPSAPQRARVAAVGAGWKAAAHAASCGAVPGEVARHHEHAAGAGQCCSAVVQAIE
APVGAVWAVVRRFDRPQAYKHFIRSCRVVDGDGGAVGSVREVRVVSGLPATSSRERLEILDDE
RRVLSFRVVGGEHRLSNYRSVTTVHEAAPAGAVVVESYVVDVPPGNTADETRTFVDTIVRCNL
QSLARTAQQLAVPA
>TaPYL7A
MRCREHDCEVPAEVARHHEHAEPGSGQCCSAVVQHVAAPAAAVWSVVRRFDQPQAYKRFVR
SCALVAGDGGVGTLREVHVVSGLPAASSRERLEILDDESHVLSFRVVGGEHRLKNYLSVTTVH
PSPAAPSSATVVVESYVVDVPAGNTTEDTRVFIDTIVKCNLQSLAKTAEKVAAVS
>TaPYL7B
MPCIPVSSPSIQHHNHNHHHRVLAGVGVGMGCGAEAVVAAAGTAGMRCREHDCEVPAEVAR

HHEHAEPGSGQCCSAVVQHVAAPAAAVWSVVRRFDQPQAYKRFVRSCALVAGDGGVGTLRE
VHVVSGLPAASSRERLEILDDESHVLSFRVVGGEHRLKNYLSVTTVHPSPAAPSSATVVVESYV
VDVPAGNTIEDTRVFIDTIVKCNLQSLAKTAEKLAAVS
>TaPYL7D
MPCIPASSPSIQHHNHNHHHRVLAGVGVGMGCGAEAVVAAAGTAGMRCREHDCEVPAEVARH
HEHAEPGSGQCCSAVVQHVAAPAAAVWSVVRRFDQPQAYKRFVRSCALVAGDGGVGTLREV
HVVSGLPAASSRERLEILDDESHVLSFRVVGGEHRLKNYLSVTTVHPSPAAPSSATVVVESYVV
DVPAGNTIEDTRVFIDTIVKCNLQSLAKTAEKVAAVS
>TaPYL8A
MVGLVGGGARAWRLSDEAANGAGGGGAATEADYMRRLHGHAPGENQCTSALVKHIKAPVH
LVWSLVRSFDQPQRYKPFVSRCVVRGGDLEIGSVREVNVKTGLPATTSTERLEQLDDDEHILSV
KFVGGDHRLRNYSSIITVHPQSIDGRPGTLVIESFVVDVPDGNTKDETCYFVEAVIKCNLTSLAE
VSERLAVQSPTSPLEQ
>TaPYL8B
MVGLLGGGARAWRLSDEAANGAVGGGAATEADYMRRLHGHAPGENQCTSALVKHIKAPVH
LVWSLVRSFDQPQRYKPFVSRCVVRGGDLEIGSVREVNVKTGLPATTSTERLEQLDDDEHILSV
KFVGGDHRLRNYSSIITVHPQSIDGRPGTLVIESFVVDVPDGNTKDETCYFVEAVIKCNLTSLAE
VSERLAVQSPTSPLEQ
>TaPYL8D
MVGLVGGGARAWRLSDEAANGAGGGGVATEADYMRRLHGHAPGENQCTSALVKHIKAPVH
LVWSLVRSFDQPQRYKPFVSRCVVRGGDLEIGSVREVNVKTGLPATTSTERLEQLDDDEHILSV
KFVGGDHRLRNYSSIITVHPQSIDGRPGTLVIESFVVDVPDGNTKDETCYFVEAVIKCNLTSLAE
VSERLAVQSPTSPLEQ
>TaPYL9A
MDGGSSGVGADGIWRPWDEHTVLRPEEMEYVRRFHQHVPGANQCTSFIAKHIKAPLQTVWS
VVRRFDKPQVYKRFVENCVMQGNIEPGCVREVTLKSGLPGKWSIERLELLDDNEHILSVMFID
GDHPLKNYSSILTVHHEVADGHPGALVIESFVVDIPKENTENEIFYLVGNFLKFNHKLLADVSEG
QIDRRALN
>TaPYL9B
MDGGSSGVGADEIWRPWDEHTVLRPEEMEYVRQFHQHEPGANQCTSFIAKHIKAPLQTVWSL
VRRFDEPQVFKPFVEKCVMQGNIEPGCVREVTIKSGLPGTWSTERLELLDDNEHILSVKFIDGD
HPLKNYSSILTVHHEVIGGHPGALVIESFVVDIPEENTENEIFYLVGNFIKINHNLLADVSERRNR
ALN
>TaPYL9D
MDGGSSGVGADGIWRPWDEHTVLRPEEMEYVRRFHQHEPGANQCTSFIAKHIKAPLQTVWS
VVRRFDKPQVYKRFVENCVMQGNIEPGCVREVTLKSGLPGKWSIERLELLDDNEHILSVKFID
GDHPLKNYSSILTVHHEVIDGHPGALVIESFVVDIPEENTKNEIFYLVGNFLKFNHKLLADVSEG
RIDRRALN

>ZmPYL1
MDQQGAGGDVEVPAGLGLTAAEYEQLRPTVDAHHRYAVGEGQCSSLLAQRIHAPPAAVWAIV
RRFDCPQVYKHFIRSCAVRPDPDAGDALRPGRLREVCVISGLPASTSTERLDHLDDAARVFGFSI
TGGEHRLRNYRSVTTVSELAGPGICTVVLESYAVDVPDGNTEDDTRLFADTVIRLNLQKLKSVA

EASTSSSAPPPPSE
>ZmPYL2
MDQQGAGGDAEVPAGLGLTAAEYEQLRSTVDAHHRYAVGEGQCSSLLAQRIHAPPEAVWAVV
RRFDCPQVYKHFIRSCALRPDPEAGDALCPGRLREVSVISGLPASTSTERLDLLDDAARVFGFSI
TGGEHRLRNYRSVTTVSELADPAICTVVLESYVVDVPDGNTEDDTRLFADTVIRLNLQKLKSV
AEANAAEAAATTNSVLLPRPAE
>ZmPYL3
MEPHMESALRQGLSEAEQRELEGVVRAHHTFPGRAPGTCTSLVTQRVDAPLAAVWPIVRGFGS
PQRYKHFIKSCDLKAGDGATVGSVREVTVVSGLPASTSTERLEILDDHRHILSFRVVGGDHRLR
NYRSVTSVTEFQPGPYCVVLESYVVDVPDGNTEEDTRMFTDTVVKLNLQKLAAIATSSSAN
>ZmPYL4
MPYTAPRPSPQQHSRVLSGGGAKAASHGASCAAVPAEVARHHEHAARAGQCCSAVVQAIAAP
VGAVWSVVRRFDRPQAYKHFIRSCRLVGGGDVAVGSVREVRVVSGLPATSSRERLEILDDERRV
LSFRVVGGEHRLANYRSVTTVHEAGAGAGTGTVVVESYVVDVPHGNTADETRVFVDTIVRCN
LQSLARTAERLA
>ZmPYL5
MPCLQASSPGSMPYQHHGRGVGCAAEAGAAVGASAGTGTRCGAHDGEVPAEAARHHEHAA
PGPGRCCSAVVQRVAAPAEAVWSVVRRFDQPQAYKRFVRSCALLAGDGGVGTLREVRVVSGL
PAASSRERLEVLDDESHVLSFRVVGGEHRLQNYLSVTTVHPSPAAPDAATVVVESYVVDVPPG
NTPEDTRVFVDTIVKCNLQSLATTAEKLALAAV
>ZmPYL6
MPCIQASSPGGMPHQHGRGRVLGGGVGCAAEVAAAVAASAGGMRCGAHDGEVPAEAARHHE
HAAAGPGRCCSAVVQHVAAPAAAVWSVVRRFDQPQVYKRFVRSCALLAGDGGVGTLREVRV
VSGLPAASSRERLEVLDDESHVLSFRVVGGEHRLRNYLSVTTVHPSPAAPDAATVVVESYVVD
VPPGNTPEDTRVFVDTIVKCNLQSLATTAEKLAAV
>ZmPYL7
MLLYMCSTSPPNPISTSPLQRETTRSIDQEDRRGSSSRPTMPYAATRTSPQQHSRVASNGRAVAA
CAGHAGVPDEVARHHEHAVAAGQCCSVMVQSIAAPADAVWSLVRRFDQPQGYKRFIRSCHLV
DGDGVEVGSVRELLVVSGLPAENSRERLEIRDDERRVISFRILGGDHRLANYRSVTTVHEAASE
GGPLTMVVESYVVDVPPGNTVEETRIFVDTIVRCNLQSLEDTVIRQQAMAAPAAPHNDHNHS
>ZmPYL8
MVGLVGGSTARAEHVVANAGGEAEYVRRMHRHAPTEHQCTSTLVKHIKAPVHLVWELVRRF
DQPQRYKPFVRNCVVRGDQLEVGSLRDVNVKTGLPATTSTERLEQLDDDLHILGVKFVGGDH
RLQVRAAAARLLRPCKNALFHAPSLLVSVLQIIFGGMLALLPFLFFLN
>ZmPYL9
MVGLVGGSTARAEHVVANAGGETEYVRRLHRHAPAEHQCTSTLVKHIKAPVHLVWELVRSFD
QPQRYKPFVRNCVVRGDQLEVGSLRDVNVKTGLPATTSTERLEQLDDDLHILGVKFVGGDHR
LQNYSSIITVHPESIDGRPGTLVIESFVVDVPDGNTKDETCYFVEAVIKCNLKSLAEVSEQLAVES
PTSPIDQ
>ZmPYL10
MVMVEMDGGVGGGGGGGGQTPAPRRWRLADERCDLRAMETDYVRRFHRHEPREHQCSSAVA
KHIKAPVHLVWSLVRRFDQPQLFKPFVSRCEMKGNIEIGSVREVNVKSGLPATRSTERLELLDD
NEHILSVRFVGGDHRLQNYSSILTVHPEVIDGRPGTLVIESFVVDVPDGNTKDETCYFVEALLK
CNLKSLAEVSERQVVKDQTEPLDR

>ZmPYL11
MVVEMDGGVGVAAAGGGGAQTPAPPPPRRWRLADERCDLRAMETDYVRRFHRHEPRDHQC
SSAVAKHIKAPVHLVWSLVRRFDQPQLFKPFVSRCEMKGNIEIGSVREVNVKSGLPATRSTERLE
LLDDDERILSVRFVGGDHRLQVCSVLHLSIFCAAHARYFAHHLKCVLEFLCQMHLDVLPCDDA
ILE
>ZmPYL12
MVGLVGGSTARAEHVVANAGGEAEYVRRMHRHAPTEHQCTSTLVKHIKAPVHLVWELVRRF
DQPQRYKPFVRNCVVRGDQLEVGSLRDVNVNPGLPATTSTERLEQLDDDLHILGVKFVGGDH
RLQLAGVVAVEVTGGPDVPFHPGREFKQIVMNMLDCFRLTVEYGVRSSQNLGDEQPQACEIK
AQLVRTAEAARQLALMLEVERPSYQGRNSVYQSSSKMMSAISVAHLGCKDMDAVDVGVVGM
VDSQSLSSALEKLYFWERKLYAEVK
>ZmPYL13
MSIPTSHIPIHIHPPTPTRPPTVFLVFTFLTQFTHPLTISPIDHCCHIPNPLPVVFGVFPAAAAAVAVV
HHTVPLAVISMRERNSSIDQEHQRGSSSRSTMPFAASRTSQQQHSRVATNGRAVAVCAGHAGVP
DEVARHHEHAVAAGQCCAAMVQSIAAPVDAVWSLVRRFDQPQRYKRFIRSCHLVDGDGAEVG
SVRELLLVSGLPAESSRERLEIRDDERRVISFRVLGGDHRLANYRSVTTVHEAAPSQDGRPLTM
VVESYVVDVPPGNTVEETRIFVDTIVRCNLQSLEGTVIRQLEIAAMPHDDNQN

>HvPYL1
MEQQPVAAAPAAEPEVPAGLGLTAAEYAQLLPTVEAYHRYAVGPGQCSSLVAQRIEAPPAAVWA
IVRRFDCPQVYKHFIRSCALRPDPEAGDDLRPGRLREVSVISGLPASTSTERLDLLDDARRAFGF
TITGGEHRLRNYRSVTTVSELSPAAPAEICTVVLESYVVDVPDGNSEEDTRLFADTVVRLNLQK
LKSVAEANAAAAAAAPPAE
>HvPYL2
MEAHMERALREGVTEAERAALEGTVRAHHTFPGRAPGATCTSLVAQRVAAPVRAVWPIVRSF
GNPQRYKHFVRTCALAAGDGASVGSVREVTVVSGLPASTSTERLEILDDDRHILSFSVVGGEH
RLRNYRSVTSVTEFQPGPYCVVVESYVVDVPDGNTEEDTRMFTDTVVKLNLQKLASVAEESG
AAPGSRRRD
>HvPYL3
PQRTQVGLLLVNCTLARERKERERKGESMEHHMESALRQGLTEPERRELEGVVEEHHTFPGRA
SGTCTSLVTQRVQAPLAAVWDIVRGFANPQRYKHFIKSCALAAGDGATVGSVREVTVVSGLPA
STSTERLEILDDDRHILSFCVVGGEHRLRNYRSVTSVTEFTDQPSGPSYCVVVESYVVDVPEGN
TEEDTRMFTDTVVKLNLQKLAAIATTTSSPPPLDGQS
>HvPYL4
MPYAAARPSPQQHSRISAACKALVAQGAAVPGEVARHHEHAAGAGQCCSAVVQAIAAPVEAV
WSVVRRFDRPQAYKRFIKSCRLVDGDGGAVGSVREVRVVSGLPGTSSRERLEILDDERRVLSFR
IVGGEHRLANYRSVTTVNEVASTVAGAPRVTLVVESYVVDVPPGNTGDETRMFVDTIVRCNLQ
SLARTAEQLALAAPRVN
>HvPYL5
MPYAAARPSPQQHSRISAACKALVAQGAAVPGEVARHHEHAAGAGQCCSAVVQAIAAPVEAV
WSVVRRFDRPQAYKRFIKSCRLVDGDGGAVGSVREVRVVSGLPGTSSRERLEILDDERRVLSFR
IVGGEHRLANYRSVTTVNEVASTVAGAPRVTLVVESYVVDVPPGNTGDETRMFVDTIVRCNLQ
SLARTAEQLALAAPRVN
>HvPYL6

MPYTASRPSAPQRARVAAGGGWKAAAHAASCGAVPGEVARHHEHAAGAGQCCSAVVQAIEA
PVGAVWAVVRRFDRPQAYKHFIRSCRLVDGDGGAVGSVREVRVVSGLPATTSRERLEILDDERR
VLSFRVVGGEHRLSNYRSVTTVHETASAGGAVVVESYVVDVPPGNTDDETRTFVDTIVRCNLQ
SLARTAQQLALAA
>HvPYL7
MPCIPASSPSIQHHNHHHRVLAGVGVGVGMGCGAEAVVAAAGTAGMRCGEHDCEVPAEVAR
HHEHAEPGSGQCCSAVVQHVAAPAAAVWSVVRRFDQPQAYKRFVRSCALVAGDGGVGTLRE
VHVVSGLPAASSRERLEILDDESHVLSFRVVGGEHRLKNYLSVTTVHPSPAAPSSATVVVESYV
VDVPAGNTIDDTRVFIDTIVKCNLQSLAKTAEKLAAVS
>HvPYL8
MDGGSSGVGADGIWRPWDEHTMLCLKEMEYVRRFHQHELGANQCTSFIAKHIKAPLQTVWS
VVRRFDKPQVFKPFVEKCVMQGNIEPGCVREVTVKSGLPAKWSIERLELLDDNEHILRVKFIDG
NHPLKNYSSILTVHHEVIDGHPGALVIESFVVDVPEENTENEIFYLVGNFLKVNHKLLADVSEG
RIDGRALN
>HvPYL9
MRSFSNPQRYKHFVRTSALVAGDEASVGNAREDTVVSGLPAFTSSERLEILDNGRHILSFSIVDG
EYCLRNYRSVSSITEFQSGPYCAVVKSYVVPDGWRHSAQQGKNLSGGFSQQDGAALIFLHFIFI
PQIQLCSHPNQCGNVFNIWELDHKYYSKTGSTPKLGDFSVFSFLLQFSSYLLPLVSASKLFGQIF
YSLLMYPHSISDAALKYDSLRCPLPLPVTMSLLLLQKMAKRSSVQAKHTISEYGKAGDRKNIT
SNAPSGPLLCFLYSWLHSTQDLFSKHVSCLDL

>SbPYL1
MVESPNPNSPSRPLCIKYTRAPARHFSPPLPFSSLIISANPIEPKAMDKQGAGGDVEVPAGLGLTA
AEYEQLRSTVDAHHRYAVGEGQCSSLLAQRIQAPPAAVWAIVRRFDCPQVYKHFIRSCALRPDP
EAGDALRPGRLREVSVISGLPASTSTERLDLLDDAARVFGFSITGGEHRLRNYRSVTTVSELAD
PGICTVVLESYVVDVPDGNTEDDTRLFADTVIRLNLQKLKSVAEANAAAAASFVSVVPPPEPEE
>SbPYL2
MEPHMETALRQGGLSELEQRELEPVVRAHHTFPGRSPGTTCTSLVTQRVDAPLSAVWPIVRGF
AAPQRYKHFIKSCDLRSGDGATVGSVREVTVVSGLPASTSTERLEILDDDRHILSFRVVGGDHR
LRNYRSVTSVTEFHHHHQAAAGRPYCVVVESYVVDVPEGNTEEDTRMFTDTVVKLNLQKLA
AIATSSAAAAASNSST
>SbPYL3
METHVERALRATLTEAEVRALEPAVREHHTFPAGRVAAGTTTPTPTTCTSLVAQRVSAPVRAVW
PIVRSFGNPQRYKHFVRTCALAAGDGASVGSVREVTVVSGLPASSSTERLEVLDDDRHILSFRV
VGGDHRLRNYRSVTSVTEFQPGPYCVVVESYAVDVPEGNTAEDTRMFTDTVVRLNLQKLAAV
AEESAAAAAAGNRR
>SbPYL4
MPCLQASSSPGSMPHQHHGRVLAGVGCAAEVAAAAVAATSPAAGMRCGAHDGEVPAEAARH
HEHAAPGPGRCCSAVVQHVAAPASAVWSVVRRFDQPQAYKRFVRSCALLAGDGGVGTLREVR
VVSGLPAASSRERLEVLDDESHVLSFRVVGGEHRLQNYLSVTTVHPSPAAPDAATVVVESYVV
DVPPGNTPEDTRVFVDTIVKCNLQSLATTAEKLAAV
>SbPYL5
MPYTAPRPSPQQHSRVTGGGAKAAIVAASHGASCAAVPAEVARHHEHAARAGQCCSAVVQAI
AAPVGAVWSVVRRFDRPQAYKHFIRSCRLVDDGGGGAGAGAGATVAVGSVREVRVVSGLPAT

SSRERLEILDDERRVLSFRVVGGEHRLANYRSVTTVHEAEAGAGGTVVVESYVVDVPPGNTAD
ETRVFVDTIVRCNLQSLARTAERLALALA
>SbPYL6
MPYAAATRTSPEQHSRVVTNGRAAVACAGHAGVPAEVARHHEHTVAAGQCCSVMMRSIAAPV
DAVWSLVRRFDQPQGYKGFIRSCHLVDGDGIEVGSVRELEVVTGLPAQNSRERLEIRDDERRVI
GFRILGGDHRLANYRSVTTVHEAASQNGGGPLTMVVESYVVDVPQGNTVEETHIFVDTIVRCN
LQSLERTVLRQQAMAAAPHNNHNHN
>SbPYL7
MVGLVGGSTARAEHVVANAGGETEYVRRLHRHAPAEHQCTSTLVKHIKAPVHLVWELVRSFD
QPQRYKPFVRNCVVRGDQLEVGSVRDVNVKTGLPATTSTERLEQLDDDLHILGVKFVGGDHR
LQNYSSIITVHPESIDGRPGTLVIESFVVDVPDGNTKDETCYFVEAVIKCNLKSLAEVSEQLAVEP
PTSPIDQ
>SbPYL8
MVEMDGGVGVVGGGQQTPAPRRWRLADELRCDLRAMETDYVRRFHRHEPRDHQCSSAVAK
HIKAPVHLVWSLVRRFDQPQLFKPFVSRCEMKGNIEIGSVREVNVKSGLPATRSTERLELLDDN
EHILSVKFVGGDHRLQNYSSILTVHPEVIDGRPGTLVIESFVVDVPDGNTKDETCYFVEALLKC
NLKSLAEVSERQVIKDQTEPLDR

Table S2. The nucleotide and amino acid sequences of PYLs identified in the genomes of three *Ipomoea* species.

Nucleotide sequences

>g928.t1 (IbPYL1)
ATGAGGGTAGGTGAGACTCCGGCGACGGAAGTGAAGATGAAGGAAGATGGATACAGCGG
CAGTGAAAGAGAGTACATAAGGAAGCACCATAGGCACCAGCCGGCAGAGAATCAATGCA
CTTCCTTTCTGATCAAGCACATCAGAGCACCTCTTCATCTCGTGTGGTCATTGGTTAGGAG
ATTTGATGAGCCACAGACGTACAAGCCATTTGTGAGGAGGTGCATTGTGCATGGAAACGT
TGAGATTGGGAGTGTTAGAGAAGTTGATGTGAGATCAGGGCTTCCCGCCACCACAAGCAC
TGAAAGATTGGAGCTCCTTGATGACGACCAGCATATCCTCAGATTCAGGATTGTTGGGGG
AGATCACAGGCTCACGAACTACTCTTCCATTCTCTCTCTCCATCCAGAGGTGGTTGATGGA
AGACCTGCAACTCTGGTGATCGAGTCGTTTGTGGTAGATGTACCTCCGGGGAACACTAAA
GATGAAACCTGCTACTTTGTACAAGCATTCATCAACTGCAATCTCAGATCTCTTGCTCACG
CTTCAGAGCAGCTATCTGAGACAACCCACTCATCATGTGTAATGGTCCAGGCAGACTCTAT
GGAACCACATCTAAGTTGCTGTTGA

>g9406.t1(IbPYL2)
ATGGTTTTGGAAGAGAAAATGGAAGCAGAGTACATAGAGAGATTCCACAAGCATCAGCCT
ACAGAGTATCAGTGTTCTTCAATTGTTACGAACCACATCAAAGCTCCCACTGATATTGTTT
GGTCACTGGTGAGGAGGTTTGATCAGCCACAGAAGTATAAGCCATTTGTTAGCAGGTGTA
CAGTTTTGGGTGATCTTGCAATTGGGAGTGTTAGAGAGGTAGATGTGAAGTCAGGACTTC
CTGCAACAACCAGCACTGAAAGGTTGGAACTTCTTGATGACGAGGAGCATATCCTTGGGA
TCAGGATCGTTGGTGGTGATCACAGACTGAAGAACTACTCTTCAGTCATTACAGTCCATCC
AGAGATAATTGATGGGAGACCAGGAACACTGGTGATCGAAGTCATTTTTGATGTCTCGGA
GAGAATGGCCATTCAGGATGGGAATGGGATTGCATCTACGACTGTGAGCTGGGCATCTAG
TGCCTATATTAAGCCGTAA

>g12068.t1(IbPYL12L)
ATGGCTGGTCACCAAAAGATTGAAGTGGACGTGGAGACTATTGAGGTTCTTGAAGGCGAT
GGAAAGGCTATTGGCTCCATCCGCTTGATCAAATATGGTGAAGGAGCTCCTCTGCTCACAT
TCGCAAAGGAGAAGATAGACGCAGTAGATGATGAGAAGAAGACAGTGAGCTACCATGTC
CTTGAGGGTGACATCCTGAAACACTACAAGCATTTCAAGGCTTTCCTCTGTGTGACTCCAA
AGGGAGATGGGAGCCTGGTGAAGTGGTGGTGCGAATTCGACAAGGCTAGTCCCGAGGTTC
CCGAACCCCACTTCATCAGGGATGCCGCCGTTAAGACATTCAAGGATCTCGAAGCCTTTCT
CAAGGCATAA

>g14758.t1(IbPYL3)
ATGAGCAGTGACCCGAACCAATACGGGTCCTCCACGACCCATCATTTGACGGTTCCACCT
GGTTTGGGTCCGGAAGAATTCGAGGAGCTGAAGCCATGGATCACCCAGTTCCACTCGTAC
CGGGTCAACTCGGGCAAATGCTCTTCCCTACTCGCACAGCGCGTCCACGCGCCGCCCGAC
ACCGTCTGGTCCGTCGTCCGCCGCTTCGACCGCCCGCAGACGTACAAGCACTTCATCAAGA
GCTGCAGCGTCGGGGAGGGGTTCCGGATGGCCGTGGGCGACACGCGTGACGTCAACGTCA
TCTCCGGACTCCCGGCGGCCACCAGCACCGAGCGCCTCGACGTCCTCGACGAGGACCGCC
GCGTGACGGGGTTCAGCATCATCGGCGGCGAGCACCGCCTCCGGAATTACCGCTCCGTCA
CTTCCGTGCATGGGATGAGCTCTAGCCCCCGCCACCCGACCACCGTCGTCTTGGAGTCCTA
CGTGGTGGACGTGCCCGAGGGAAATACGGAGGAGGACACCAAGCTCTTCGCCGACACCGT
CGTCAGGTTGAACCTCCAGAAGCTGGCCTGCATCACCGAAGCCATGGCGGGAAGGTGGCG

GCGGCACCGCCGCCGCCGCCGGCGACACAAGTCATCATTGAGGGTACGGTGA
>g19566.t1(IbPYL4)
ATGCTCGGCGGCGATAGGAGGACCGGTGTGGAGGACGAGTACATCCGGAGACACCACAG
ACATGAGGTCAGAGACAACCAGTGTAGCTCCTCGCTCGTTAAGCACATCAGAGCTCCGGT
TCATCTCGTGAGTCTCCTCCGCACGCTTCTCTTTTGTTCTTCCGATTTTCTGTTCGTTTCTGC
TCAGTATTACTCCGATCTGCTCGTTCCTGCTACTGATTGCGATCCTTTTGAATTTACACCGT
TGACCATGGATGTTTGGATATTTGATGAAGTATGGTCGTTAGTGAGGAGATTTGATCAACC
ACAAAGGTATAAACCATTTGTGAGCAGGTGCATCGTGCAGGGAGACCTTGAAATTGGAAG
TGTTAGAGAAGTTAATGTTAGGTCAGGACTTCCGGCAACCACAAGCAAGGAGAGACTAGA
GCTCCTAGATGATAATGAACATATCTTTAGCATGAGGATTGTTGGTGGCGATCACAGGCTA
AGGAATTACTCATCAATAATAACCGTTCATCCAGAGATCATCGACAGCAGGCCTGGGACA
TTGGTAATTGAGTCATTTGTTGTGGACGTTCCTGATGGGAACACCCAGGATGAAACGTGCT
ACTTTGTGGAGGCTCTGATCAGGTGCAACCTCAAGTCTCTGGCTGAGGTCTCAGAACGATT
GGCAGTGCAGGGCCATACAGAGCCCATCGACAGAATCTGA
>g20407.t1(IbPYL5)
ATGCCTACTACAGTTCACTGCCATATATCCGATTCGCACCACCACCTCTCCGCCGCTGTCT
CGGCGGCGACGACGGCGGTGAGGATCCAGAAGCAGTCGCCGTTGCCGACATGGACGGTG
GTGGTCCCGGACAGCTTGCTGCAGTACCACGTCCACGCGGTGGGGCCCAACCAGTGCTGT
TCCGCGGTGGTGCAGGAGGTGTCAGCCCCGCTGGACGCCGTGTGGGCCCTCGTCCGCCGC
TTCGACAACCCGCAGGCCTACAAGCATTTCCTCAAGAGCTGCCACGTCATCGTCGGGGAC
GGCAGCAGCGTGGGCACGCTCCGCGAGGTGCGCGTGGTGTCCGGCGTCCCGGCCGTCTCC
AGCACCGAGCGCCTCGATATCCTCGACGACGAGCGACACGTGTTCGGCTTCAGCGTCGTC
GGCGGCGACCACCGCCTCCGCAACTACCGCTCCGTCACCACTCTCCACCACCGCGCCGCC
GCAGATTCCGAACACCACAAGACGGTCGTCGTGGAATCCTTCGTCGTCGACGTCCCGCCG
GGGAATACTAAGGAAGAGACGTGCGTCTTCGTTGACACCATCGTGCGGTGCAATCTCCAG
TCGTTAGCGCAAATCGCGGAGAACTCCGGCAAGAATAATCAAAACATCGTCAAATCTTCT
TGTAATTAA
>g20643.t1(IbPYL6)
ATGAACGCGGCTGGGAACGGCGGTGTGGAGAGAGAGTACATAACGAAACATCACAGGCA
CGCGCCGGCGGAGAATCAATGCAGTTCGTTTCTGATCAAGCACATCAGAGCACCTGTTAA
CCTCGTTTGGTCATTGGTTAGGAGGTTTGATCAGCCACAGAAATACAAGCCCTTTATCAGC
AGGTGCATTAGTCAGGGAAGTCTTGAGATTGGTAGTCTTAGAGAAGTTGATGTCAAGTCA
GGCCTGCCTGCCACAACAAGCACAGAAAGGTTGGAGCTTCTTGATGATGATGAGCATATA
CTCAGTGTGAGGATTGTTGGTGGGGATCACCGACTTCGGAACTATTCCTCAGTCATATCTG
TCCATCCAGAGGTCATTGACGGAAGACCTGGAACTGTGGTGATAGAATCATTTGTGGTAG
ATGTGCCAGAAGGAAACACTATCGACGAAACCTGCTACTTTGTTGAAGCATTAATCAAAT
GCAATCTGAAATCTCTTGCGGATGTTTCAGAGCGGCTGGCTGTGCAGGGCAGGACAGAAC
CCATAGATCCTGTGTAA
>g24506.t1(IbPYL7)
ATGTCTGAAATGCTTCCGAATCCGCCGGAGAGGTCTGTTTTGCTTCAAAGACTAATCCCGG
GGGCGGGCGCCACGTGTAAGCAATCGCAGCCGCAGTATCAACGGCGGACGCCGATTCCCT
GCGACAGCCATGTACCGGAGACGGCGGCGCGGTATCACGCGTATTCGGTGAGTCCCAACC
AGTGCTGCTCCGCCGTGATCCAGTACATCGCGGCCCCGGTGTCCGCCGTGTGGGGCGTGGT
GCGGCGCTTCGATAACCCGCAGGCGTACAAGCACTTCGTGAAGAGCTGCCACGTCATCGC

GGGGGAAGGGGACGTGGGGACGCTCCGCGAGGTGCGCGTGGTGTCCGGACTCCCCGCGG
CCACCAGCACGGAGCGGTTAGAGATCCTGGACGAGGAGCGCCACGTCATCAGCTTCAGCG
TCGTCGGCGGGGACCACCGCCTGGCGAACTACCGATCCGTCACGACGCTCCACGCGGGGG
CGGGCGGCGGAGACGGCGAGGAGGAGGACGAGGACGGCACCGTCGTGGTGGAGTCGTAC
GTGGTCGACATCCCGCCGGGGAACACGCGGGAGGAGACGTGCGTGTTCGTCGACACTATC
GTGAAATGCAACCTCCAGTCGCTGGCGCAGATCGCCGAGAGCTCCGGCCGGCGAAACTCG
GCTAGCTTGAATTGA
>g25480.t1(IbPYL8)
ATGCCTTCTGCGCTTCAGCTTCACCGATCAAATCAGCTCGGCTCGGCGGCGGTGGCGGCGG
CGAGTGTTTATAAGCAGGCGGCGGCGGCGCAGGCGGCTAGGTGGATGATTCCGGTGTCTA
TATCGGTGCCGGAGCACGTGCTGCAGTACCACACGCACGCGGTGGGGGCGGGGCAGTGCT
GCGCGGCGGTGGTGCAGGAGGTGGCGGCGCCGCTGGAGGCGGTGTGGCGGCTGGTGCGC
CGCTTCGACAAGCCGCAGGCGTACAAGCACTTCCTGAAGAGCTGCCACGTCATTGTCGGC
GACGGGGACGTGGGCACGCTCCGCGAGGTGCGCGTGGTGTCCGGCCTCCCCGCCGCCTCC
AGCACCGAGCGCCTCGAGATCCTCGACGACGAGAAGCACGTGCTCAGCTTCGCCGTCGTC
GGCGGCGATCACCGCCTCAACAACTACCGCTCCGTCACCACCCTCCACCCCACCACCGCA
ACAACACCACCGTCGTCGTCGAGTCCTACGTCGTCGACGTCCCCCCCGGCAACACCAACG
ACGAAACCTGCGTCTTCGTCGACACCATCATCAAGAACTGAAAAGTCATACTACCAATAC
AGTGATAGATGTTCGTTGAAGATTATAAAGCTAAGGTTCGTTATTAGATTTGGAGGGTGTG
ATGAAGATCTCGTATGA
>g26930.t1(IbPYL9)
ATGAACGCGAACGGATACAGCAGTGTTGAAAGAGAGTACATAAGGAAACATCATAGGCA
AGAGCCGGCGGGGAATCAATGCAGTTCGTTTCTGATCAAGCACATTAGAGCGCCTGTTCA
TCTCGTTTGGTCATTGGTTCGGAGGTTTGATCAACCACAGAAGTACAAGCCCTTTGTCAGC
CGGTGCATAGCACAGGGAAACCTTGAGATTGGTAGTCTTAGAGAAGTTGATGTCAAATCA
GGCCTTCCTGCCACAACAAGCACGGAAAGGTTAGAACTTCTGGATGATGATGAACATATC
CTCAGCGTGAAGATTGTTGGGGGAGATCACAGACTTAGGAACTACTCCTCTGTCATATCTG
TCCATCCAGAAGTAATTGATGGACGACCCGGAACTCTGGTTATTGAATCATTTGTGGTAGA
TGTGCCTGAAGGAAACACTAAAGATGAGACCTGCTACTTTGTTGAAGCATTGATCAAGTG
CAATCTGAAATCTCTAGCCGATGTTTCCGAGAGGCTTGCTTTGCAGGGCAGGACAGAGCC
CATTGATCAGGTGTAG
>g39321.t1(IbPYL10)
ATGACGGCCGACATGATGAGCGCCGATAGAAGAACCGACGTAGAGGATGAGTACATTAG
GAGGCACCACAAGCACGAGGTCAGGGAGAACCAGTGTAGTTCCTGTCTTGTAAAGCACAT
CAGAGCTCCAGTTCATCTTGTTTGGTCATTGGTAAGGAGGTTTGATCAGCCTCAGAGGTAC
AAACCGTTTGTGAGCAGGTGTATTGTGCAGGGCGACCTTGAAATTGGAAGTGTTAGAGAA
GTTAATGTTAAGTCTGGCCTTCCAGCCACCACCAGCAAGGAGAGGCTAGAGCTGCTGGAT
GACGACGAGCACATATTTAGCATGAGGATTGTTGGTGGAGATCACAGGCTAAGGTTCAAT
TACTTGATACTAGTGGACGTTGTGTGTTCTGTGTTGCATTTGCCTGAACACTTTGGCCTTTT
AGGCTGTGGCTGTTCTTTGAAATGGCAGTGTGGTTGGCTTAGGAATTTGATCTGCTATTTT
CTTTTCGTACTTCTTGCTCGCAAGGATTACCGTAGAATTTCACAGGCTGTTCTTAAGTTTCA
TCCCGAGTCCCAACTTCTAATTCTTTCTCATCCCGCATTTAAAAACATGCAGAACTACTCA
TCAATCGTGACTGTACACCCAGAGATCATTGATGGCAGGCCTGGAACATTGGTCATAGAA
TCATTTGTGGTGGACATCCCAGACGGGAACACCAAGGATGAAACATGCTACTGTGGAGGC

GCTGATTAA
>g55788.t1(IbPYL11)
ATGGAGAAAGCTGAGAGCTCGATGAACAGTGATTTGCAGCGAGGAGAAGATGGAGAAGT
CGGGTCGGAGTCGACCCATCACTTGACGGTTCCGCCCGGATTGAACCCGGACGAGTTCGA
GGAGCTGAGGCCGTGGGCGATTGAGTTCCACACCTACCGAGTCAACTCGGGCCAATGCTC
CTCCCTGCTGGCGCAGCGAATCCACGCGCCGCCGGACACGGTGTGGTCCGTGGTTCGCCA
GTTCGACAAGCCGCACACCTACAAGCACTTCATCAAGAGCTGCACCGTGTGCGAGGGGTT
CCGGATGGCGGTGGGCGACACGCGCTACGTCAACGTCATCTCCGGCCTGCCGGCGGCCAC
CAGCACCGAGCGCCTCGACATCCTCGACGACGGCCGCCGCGTCACGGGCTTCAGCATCAT
TGGCGGCGAGCACCGCCTGAGGAACTACCGCTCCGTCACCTCCGTCCACGGCCTCCGCCG
CCGCGACGGCGGCCGGAGCTGCACCGCCGTGCTGGAATCCTACGTGGTGGACGTGCCGGA
GGGGAACACGGAGGAGGACACCAAGCTTTTCGCCGACACCGTCGTTAAGCTCAACCTTCA
AAAACTCGCATCCATCACCGAAGGGATCAATCAGGACAGAAACAACGATGCTAATGAAG
AAGATAGTGGTAATAATACAACCGAACGTCACACTTGA
>g55782.t1(IbPYL13L)
ATGCTCCTCCCTGCTGGCGCAGCGAATCCACGCGCCGCCGGACACGGTGTGGTCCGTGGTT
CGCCAGTTCGACAAGCCGCACACCTACAAGCACTTCATCAAGAGCTGCACCGTGTGCGAG
GGTTCCGGATGGCGGTGGGCGACACGCGCTACGTCAACGTCATCTCCGGCCTGCCGGCGG
CCACCAGCACCGAGCGCCTCGACATCCTCGACGACGGCCGCCGCGTCACGGGCTTCAGCA
TCATTGGCGGCGAGCACCGCCTGAGGAACTACCGCTCCGTCACCTCCGTCCACGGCCTCCG
CCGCCGCGACGTGGGCCGAGCTGCACCGCCGTGCTGGAATCCTACGTGGTGGACGTGCCG
GAGGAACACGGAGGAGGACACCAAGCTTTTCGCCGACACCGTCGTTAAGCTCAACCTTCA
AAAACTCGCATCCATCACCGAAGGGATCAATCAGGACAGAAACAACGATGCTAATGAAG
AAGATAGTGGGAATGGTGATAAGGAAATGTTGGCCTGGAGTGATCATTTTGTACAAGTTT
TGAACAACAACGATGTTGGGATGATGGTTTTATAA

>itf02g00240.t1
ATGAGCAGTGACCCGAACCAATACGGGTCCTCCACGACCCATCATTTGACGGTACCACCC
GGTTTGGGTCCGGAAGAATTCGAGGAGCTGAAGCCCTGGATCACCCAGTTCCACTCGTAT
CGGGTCAACTCGGGCAAATGCTCTTCCCTACTCGCACAGCGCGTCCACGCGCCGCCCGAC
ACCGTCTGGTCCGTCGTCCGCCGCTTCGACCGCCCGCAGACGTACAAGCACTTCATCAAGA
GCTGCAGCGTCGGGGAGGGGTTCCGGATGGCCGTGGGCGACACGCGTGACGTCAACGTCA
TCTCCGGCCTCCCGGCCGCCACCAGCACCGAGCGCCTCGACGTCCTCGACGAGGACCGCC
GCGTGACGGGGTTCAGCATCATCGGCGGCGAGCACCGCCTCCGGAACTACCGCTCCGTCA
CTTCCGTGCATGGGCTGAGCTCTAGCCCCCGCCACCCGACCACCGTCGTCTTGGAGTCCTA
CGTGGTGGACGTGCCCGAGGGAAACACCGAGGAGGACACCAAGCTCTTCGCCGACACCGT
CGTCAGGTTGAACCTCCAGAAGCTGGCCTGCATCACCGAAGCCATGGCGCGAGGGGAAGG
TGGCGGGGGCGCCACCGCCGCCGCCGCCGGCGACACAAGTCATCATTGA
>itf03g25530.t1
ATGTTTCTGTATAGAAACGGATTCGGAGGAGTGGTATTGAATAGAGAGAGAGAAGCAACG
ATGAACGCGAACGGATACAGCAGTGTTGAAAGAGAGTACATAAGGAAACATCATAGGCA
AGAGCCGGCGGGGAATCAATGCAGTTCGTTTCTGATCAAGCACATTAGAGCGCCTGTTCA
TCTCGTTTGGTCATTGGTTCGGAGGTTTGATCAACCACAGAAGTACAAGCCCTTTGTCAGC
CGGTGCATAGCACAGGGAAACCTTGAGATTGGTAGTCTTAGAGAAGTTGATGTCAAATCA

GGCCTTCCTGCCACAACAAGCACGGAAAGGTTAGAACTTCTGGATGATGATGAACATATC
CTCAGCGTGAAGATTGTTGGGGGAGATCACAGACTTAGGAACTACTCCTCTGTCATATCTG
TCCATCCAGAAGTAATTGATGGACGACCCGGAACTCTGGTTATTGAATCATTTGTGGTAGA
TGTGCCTGAAGGAAACACTAAAGATGAGACCTGCTACTTTGTTGAAGCATTGATCAAGTG
CAATCTGAAATCTCTAGCCGATGTTTCCGAGAGGCTTGCCTTGCAGGGCAGGACAGAGCC
CATTGATCAGGTGTAG
>itf03g14430.t1
ATGCCTTCTGCGCTTCAGCTTCACCGATCAAATCAGCTCGGCTCAGCGGCGGCGGCGGCG
GCGACGAGTGTTTATAAGCAGGCGGCGGCGGCGCAGGCGGCTCGGTGGATGATTCCGGTG
TCGATATCGGTGCCGGAGAACGTGCTACAGTACCACACGCACGCGGTGGGGGCGGGGCA
GTGCTGCGCGGCGGTGGTGCAGGAGGTGGCGGCGCCGCTGGAGGCGGTGTGGCGGCTGGT
GCGCCGCTTCGACAAGCCGCAGGCGTACAAGCACTTCCTGAAGAGCTGCCACGTCATTGT
CGGCGACGGGGACGTGGGCACGCTCCGCGAGGTGCGCGTGGTGTCCGGCCTCCCCGCCGC
CTCCAGCACCGAGCGCCTCGAGATCCTCGACGACGAGAAGCACGTGCTCAGCTTCGCCGT
CGTCGGCGGCGATCACCGCCTCAACAACTACCGCTCCGTCACCACCCTCCACCCCCACCAC
CGCAACAACACCACCGTCGTCGTCGAGTCCTACGTCGTCGACGTCCCCCCCGGCAACACC
AACGACGAAACCTGCGTCTTCGTCGACACCATCGTACGTTGCAATCTTCAATCCCTAGCGC
AAATCGCGGAAAACTCCAACAACTCTCCAAATCAAGAACTGAAAAGTCATACTACCAATA
CAGTGATAGATGTTCGTTGA
>itf04g00430.t1
ATGGTTTTGGAAGAGAAAATGGAAGCAGAGTACATAGAGAGATTCCACAAGCATCAGCCT
ACAGAGTATCAGTGTTCTTCCATTGTTACGAACCACATCAAAGCTCCCACTGATATTGTTT
GGTCACTGGTGAGGAGGTTTGATCAGCCACAGAAGTATAAGCCATTTGTTAGCAGGTGTA
CAGTTTTGGGTGATCTTGCAATTGGGAGTGTTAGAGAGGTAGATGTGAAGTCAGGACTTC
CTGCTACAACCAGCACTGAAAGGTTGGAACTTCTTGATGATGAGGAGCATATCCTTGGGA
TCAGGATTGTTGGTGGTGATCACAGACTGAAGAACTACTCTTCAGTCATTACAGTCCATCC
AGAGATAATTGATGGGAGACCAGGAACACTGGTGATCGAATCATTTTTGGTAGATGTGCC
TGAAGGGAACACTCGAGACGATACCTGCTACTTTGTGAATGCGCTTATCAACTGTAACCTG
AAAGCTTTGGCAGATGTCTCGGAGAGAATGGCCATTCAGGATGGGAATGGGATTGCATCT
ACGACTGTGAGCTGGGCATCTAGTGCCTATATTAAGCCGTAA
>itf05g19440.t1
ATGGAGCAGAGGCTTATTGTACCTTTGAATGAGCAGAGCGGAGCAGGCAGCAGATGCATG
AGGACTGCCACGTTGCAACTTTCTCTTCGGTCACTACTCACTTCCAAAGCTTCAACAGCCA
GGCGTCTCTCTCGTGTATCTGAATGGTATGCACTAGTTGCTTGGGATCTGTGGTTGTTGAA
GCTGGACAATTTGGGATTTTTCAAGCTCCACGGATTAGTTACAACAAGAAGAAAGATGAT
GGCAGATACTAGTTCAATGGCTAAACAAGGACCAGTTCTTGATGTTAAACCTGTGAAGGA
TGATGACGGTGGCTATGCCAGTGGAGGATGGAAGAGTGAAGATGGAAGGTTGAGTTGTG
GTTATTCTAGCTTCAGAGGAAAGAGAGCAAGCATGGAGGACTTTTATGATATTAAGACTT
CCAAGATTGATGGAAAGACAATTTGTTTGTTTGGGATATTTGATGGCCATGGGGGTTCACG
TGCAGCAGAGTATTTAAAGCAAAATCTCTTTGAGAATCTGATGAAGCATCCAGAGTTCGT
AACAAACACTAAACATGCTATAAGTGAAACATATCAACAGACTGATAGGGATTTTTTGGA
GTCTGAAAAAGATTCTCTCCGTGATGATGGTTCTACTGCATCAACAGCAGTTCTGGTTGGG
AATCATCTATATGTTGCCAACGTTGGCGATTCAAGAACCATTGTGTCAAAAAAAGGAAAA
GCAATTCCTCTCTCTCAGGATCATAAACCAAATAGAAGTGATGAGAGAAAGAGGATTGAA

AATGCTGGAGGTGTTATTATGTGGGCAGGCACTTGGAGAGTTGGAGGAGTCTTGGCAATG
TCCCGTGCCTTTGGTAATCGTATGCTGAAGCAATATGTTGTGGCTGAACCTGAGATTCAGG
AAGAAGTCATTGATGAAGAATTAGAATTGCTTGTCCTGGCAAGTGATGGACTCTGGGATG
TAGTACCCAATGAGGATGCCGTTTCACTAGCAGAGTCGGAAGAATCACCCGAAGCGGCTG
CGAGGAAGCTGACAGAGACTGCTTTTACCCGTGGCAGTGCTGACAATATCACCTGCATCG
TGGTGAAGTTCCATCACAAGAAGGCAGAGCCTGAGTGGGAGTGGGAGTGGGAGTGGGAG
ATGAGGGTAGGTGAGACTCCGGCGACGGAAGTGAAGATGAAGGAAGATGGATACAGCGG
CAGTGAAAGAGAGTACATAAGGAAGCACCATAGGCACCAGCCGGCAGAGAATCAATGCA
CTTCCTTTCTGATCAAGCACATCAGAGCACCTCTTCATCTCGTGTGGTCATTGGTTAGGAG
ATTTGATGAGCCACAGACGTACAAGCCATTTGTGAGGAGGTGCATTGTGCATGGAAACGT
TGAGATTGGGAGTGTTAGAGAAGTTGATGTGAGATCAGGGCTTCCCGCCACCACAAGCAC
TGAAAGATTGGAGCTCCTTGATGACGACCAGCATATCCTCAGATTCAGGATTGTTGGGGG
AGATCACAGGCTCACGAACTACTCTTCCATTCTCTCTCTCCATCCAGAGGTGGTTGATGGA
AGACCTGCAACTCTGGTGATCGAGTCGTTTGTAGTAGATGTACCTCCGGGGAACACTAAA
GACGAAACCTGCTACTTTGTACAAGCATTCATCAACTGCAATCTCAGATCTCTTGCTCACG
CTTCAGAGCAGCTATCTGAGACAACCCATTCATCATGTGTAATGGTCCAGCCAGACTCTAT
GGAACCACATCTAAGTTGCTGTTGA
>itf06g09740.t1
ATGGAGAAGACGGTCCAAATCCCTCAAGGGCTAACGGAAGAGGAATATTCCCAGCTGGA
AACGGTGATCCAATCTTACCACACCTTCGACCCCAGACCCAACACGTGCACGTCCCTGATA
ACGCAGCACATCGACGCGCCGGCGAGCGTGGTGTGGCCGTTCGTCCGCCGCTTCGACAAC
CCGCAGAAGTACAAGCACTTCATCAAGAGCTGCCGGATGGTCTCCGGCGACGGCGGCGTG
GGGAGCGTTCGGGAGGTCACCGTGGTCTCCGGCATCCCCGCGTCCACCAGCACCGAGCGC
CTGGAGATCCTCGACGACGACAAGCGAATCATAAGCTTCCGAGTGGTCGGCGGCGAGCAC
CGGCTGAACAACTACCAGAGCGTCACTTCCGTCAACGAGTTCCAGAAGAAGAATGGGAGC
GTTTACACGGTGGTTTTGGAGTCGTATATCGTTGATATACCGGAAGGGAATACGGTGGTG
GACACGAAGATGTTCACCGATACCGTCGTCAAGCTCAACCTGCAGAAGCTTTGCATGGTG
GCAATGGCCGCTTTGCATGGCCACGAATAA
>itf08g08150.t1
ATGACGGCCGACATGATGAGCGCCGATAGAAGAACCGACGTAGAGGATGAGTACATTAG
GAGGCACCACAAGCACGAGGTCAGGGAGAATCAGTGTAGTTCCTGTCTTGTAAAGCACAT
CAGAGCTCCAGTTCATCTTGTTTGGTCATTGGTAAGGAGGTTTGATCAGCCTCAGAGGTAC
AAACCGTTTGTGAGCAGGTGTATTGTGCAGGGCGACCTTGAAATTGGAAGTGTTAGAGAA
GTTAATGTTAAGTCTGGCCTTCCAGCCACTACCAGCAAGGAGAGGCTAGAGCTGCTGGAT
GACGACGAGCACATATTTAGCATGAGGATTGTTGGTGGAGATCACAGGCTAAGGAACTAC
TCATCAATCGTGACTGTACACCCAGAGATCATTGATGGCAGGCCTGGAACATTGGTCATA
GAATCATTTGTGGTGGACATCCCAGACGGGAACACCAAGGACGAAACATGCTACTTCGTG
GAGGCGCTGATTAAGTGCAACCTCAAGTCGTTGGCTGATGTCTCGGAGCGATTGGCAGTA
CAGGGCCGCACGGAGCCTATTGACAGAGTGTAG
>itf09g25130.t1
ATGCCACCTACAATAGTTACTGTTGAGCTCCGAATCCACGCGCCGCCGGACACGGTGTGG
TCCGTGGTTCGCCAGTTCGACAAGCCGCACACCTACAAGCACTTCATCAAGAGCTGCACC
GTGTGCGAGGGGTTCCGGATGGCGGTGGGCGACACGCGCTACGTCAACGTCATCTCCGGC
CTCCCGGCGGCCACCAGCACCGAGCGCCTCGACATCCTCGACGACGGCCGCCGCGTCACG

GGCTTCAGCATCATTGGCGGCGAGCACCGCCTGAGGAACTACCGCTCCGTCACCTCCGTCC
ACGGCCTCCGCCGCCGCGACGGCGGCCGGAGCTGCACCGCCGTGCTGGAATCCTACGTGG
TTGACGTGCCGGAGGGGAACACGGAGGAGGACACCAAGCTCTTCGCCGACACCGTCGTCA
AGCTCAACCTTCAAAAACTCGCATCCATCACCGAAGGGATCAATCAGGACAGAAACAACG
ATGCTAATGAAGAAGATAGTGGTAATAATACAACCGAACGTCACACTTGA
>itf12g23670.t1
ATGCCTACTACAGTTCACTGCCATATATCCGATTCGCACCACCACCTCTCCGCCGCTGTCG
CGGCGGCGACGACGGCGGTGAGGATCCAGAAGCAGCCGCCGTTGCCGACGTGGACGGTG
GTGGTCCCGGACAGCTTGCTGCAGTACCACGTCCACGCGGTGGGGCCCAACCAGTGCTGT
TCCGCGGTGGTGCAGGAGGTGTCCGCCCCGCTGGACGCCGTGTGGGCCCTCGTCCGCCGC
TTCGACAACCCGCAGGCCTACAAGCATTTCCTCAAGAGCTGCCACGTCATCGTCGGGGAC
GGCAGCAGCGTGGGCACGCTCCGCGAGGTGCGCGTGGTGTCCGGCGTCCCGGCCGTCTCC
AGCACCGAGCGCCTCGATATCCTCGACGACGAGCGACACGTGTTCGGCTTCAGCGTCGTC
GGCGGCGACCACCGCCTCCGCAACTACCGCTCCGTCACCACTCTCCACCACCGCGCCGCC
GACGACCACGAACACAACAAGACGGTCGTCGTGGAATCCTTCGTCGTCGACGTCCCGCCG
GGGAATACTAAGGAAGAGACGTGCGTCTTCGTTGACACCATCGTGCGGTGTAATCTCCAG
TCGTTAGCGCAAATCGCGGAGAACTCCGGCAAGAATAATCAAAACATCGTCAAATCTTCT
TGTAATTAA
>itf12g11260.t1
ATGGCTCCCAACCCACCAAAGACTTCCGTTCTACTTCAAAGAATCAGCACTCGCAAGCAA
TCTCAACGGCGCTCATCGCCGATAATATCTTGCGCCGCCGCGGCCTCGGAGGTCCCGGAA
GCGGTGGCCCGCTATCACGCCCATCCGGTGGGACCCAACCAGTGCTGCTCCGCCGTGATC
CAGCGGATCTCCGCCCCCGTCTCCACCGTTTGGTCCGTGGTCCGCCGCTTCGACAACCCGC
AGGCCTACAAGCACTTCGTGAAGAGCTGCGACGTCATCGTGGGGGACGGCGACGTGGGCA
CCCTCCGCGAGGTCCGCGTCATCTCCGGCCTCCCCGCCGGCAGCAGCACCGAGCGGCTCG
AGATCCTCGACGAGGAGCGCCACGTCATCAGCTTCAGCGTCGTCGACGGCGACCACCGCC
TCGCCAACTACCGCTCCGTCACCACCCTCCACGCGGACGCGGGCGGGACGACCACCGTCG
TGGAGTCCTACGTTGTTGATGTCCCGCCGGGAAACACGAGAGACGAAACCTGCGTCTTCG
TCGACACTATCGTCAAATGTAACCTCCAGTCGCTGGCGCAGATCGCCCAGAATTCCGGCC
GACGAAACAACAACTGA
>itf12g25980.t1
ATGAACGCGGCTGGGAACGGCGGTGTGGAGAGAGAGTACATAACGAAACACCACAGGCA
CGCGCCGGCGGAGAATCAATGTAGTTCGTTTCTGATCAAGCACATCAGAGCACCTGTTAA
CCTCGTTTGGTCATTGGTTAGGAGGTTTGATCAGCCACAGAAATACAAGCCCTTTATCAGC
AGGTGCATTAGTCAGGGAAGTCTTGAGATTGGTAGTCTTAGAGAAGTTGATGTCAAGTCG
GGCCTGCCTGCCACAACAAGCACAGAAAGGTTGGAGCTTCTTGATGATGATGAGCATATA
CTCAGTGTGAGGATTGTTGGTGGGGATCACCGACTTCGGAACTATGCCTCAGTCATATCTG
TCCATCCAGAGGTCATTGACGGAAGACCAGGAACTGTGGTGATAGAATCATTTGTGGTAG
ACGTTCCAGAAGGAAACACTATTGACGAAACCTGCTACTTTGTTGAAGCATTAATCAAAT
GCAATCTGAAATCTCTTGCGGATGTTTCAGAGCGGCTTGCTGTGCAGGGCAGGACAGAAC
CCATAGATCCTGTGTAA
>itf12g17820.t1
ATGACGGTCAGCATGCTCGGCGGCGATAGGAGGACCGGTGTCGAGGACGAGTACATCCG
GAGACACCACAGACATGAGGTCAGAGACAATCAGTGTAGCTCCTCGCTCGTTAAGCACAT

CAGAGCTCCGGTTCATCTCGTATGGTCGTTAGTGAGGAGATTTGATCAACCACAAAGGTAT
AAACCATTTGTGAGCAGGTGCATCGTGCAGGGAGACCTTGAAATTGGAAGTGTTAGAGAA
GTTAATGTTAGGTCAGGACTTCCGGCAACCACAAGCAAGGAGAGACTAGAGCTCCTAGAT
GATAATGAACATATCTTTAGCATGAGGATTGTTGGTGGCGATCACAGGCTAAGGAATTAC
TCATCAATAATAACCGTTCATCCAGAGGTCATCGACAGCAGGCCTGGGACATTGGTAATT
GAGTCATTTGTCGTAGACGTTCCTGATGGGAACACTCAGGATGAAACGTGCTACTTTGTGG
AGGCTCTGATCAAGTGCAACCTCAAGTCTCTGGCTGAGGTCTCGGAACGACTGGCAGTGC
AGGGCCATACAGAGCCCATCGACAGAATCTGA
>itf15g06620.t1
ATGTCTGAAATGCTTCCGAATCCGCCGGAGAGGTCTGTTTTGCTTCAAAGACTCATCCCGG
GGGCGGGCGCCACGTGTAAGCAATCGCAGCCTCAGTATCAACGGCGGACGCCGATTCCCT
GCGACAGCCATGTGCCGGAGACGGCGGCGCGGTATCACGCGTATTCGGTGAGTCCCAACC
AGTGCTGCTCCGCCGTGATCCAGTACATCGCGGCCCCGGTGTCCGCCGTGTGGGGCGTGGT
GCGCCGCTTCGATAACCCGCAGGCGTACAAGCACTTCGTGAAGAGCTGCCACGTCATCGC
GGGGGAGGGCGACGTGGGGACGCTCCGTGAGGTGCGCGTGGTGTCCGGCCTCCCCGCGGC
CACCAGCACGGAGCGGTTAGAGATCCTGGACGAGGAGCGCCACGTCATCAGCTTCAGCGT
CGTCGGCGGGGACCACCGCCTGGCGAACTACCGATCCGTCACGACGCTCCACGCGGGGGC
GGGCGGCGGAGACGGCGAGGACGAGGACGGCACCGTCGTGGTGGAGTCGTACGTGGTCG
ACATCCCGCCGGGGAACACGCGGGAGGAGACGTGCGTGTTCGTCGACACTATCGTGAAAT
GCAACCTCCAGTCGCTGGCGCAGATCGCCGAGAGCTCCGGCCGGCGAAACGCGGCTAGCT
TGAATTGA
>itf15g20740.t1
ATGGCGGAGAGAGTCCAAATCCCGCAGGGGCTAACGGAGGAGGAATTCTCGGAGCTAGA
GCCGTTGATCCGGCGGTACCACACCTTCGATCACGTGCCCAACACATGCACGTCTCTGATC
ACGCAGCACATCGACGCGCCGGCGAGCGTGGTGTGGCCCATCGTCCGCCGCTTCGACAAC
CCGGAGAAGTACAAGCATTTCATCAAGAGCTGCCGGATGACCGGCGACGGCGGCGTGGG
GAGCATCCGGGAAGTCACCGTCGTCTCCGGCATCCCCGCCGCCACCAGCACCGAGCGCCT
CGAGATCCTCGACGACCACAACCACATCCTCAGCTTCCGCGTCGTCGGCGGCGAGCACCG
CCTCAATAACTACCGGAGCGTCACCTCGGTCAACCAATTCCCCCGCCGCCGCGGGGCCCA
CAATAACAATAACAATAATAATGACTTTTACACAGTCGTTTTGGAGTCTTATATCGTCGAT
ATACCCGAAGGGAACACCGGAGCCGATACCCGGATGTTTACCGACACGGTGGTTAAGCTG
AATCTGCAGAAGCTTGGGGTGGTGGCTATGGCGGCTTTGCATGGCGGAGGCGGAGGCGGA
GGTAGTGGTGGTCACTCATGA

>itb13g12810.t1
ATGAGCAGTAACCCGAACCAATACGGGTCCTCCTCGACCCATCATTTGACGGTTCCACCCG
GTTTGGGTCCGGAAGAATTCGAGGAGCTGAAGCCATGGATCACCCAGTTCCACTCGTACC
GGGTCAACTCGGGCAAATGCTCTTCCCTACTCGCACAGCGCGTCCACGCGCCGCCCGACA
CCGTCTGGTCCGTCGTCCGCCGCTTCGACCGCCCGCAGACGTACAAGCACTTCATCAAGAG
CTGCAGCGTCGGGGAGGGGTTCCGGATGGCCGTGGGCGACACGCGTGACGTCAACGTCAT
CTCCGGCCTCCCGGCCGCCACCAGCACCGAGCGCCTCGACGTCCTCGACGAGGACCGCCG
CGTGACGGGGTTCAGCATCATCGGCGGCGAGCACCGCCTCCGGAACTATCGCTCCGTCAC
TTCCGTGCATGGGCTGAGCTCTAGTCCCCGCCACCCGACCACCGTCGTCTTGGAGTCCTAC
GTGGTGGACGTGCCCGAGGGAAATACGGAGGAGGACACCAAGCTCTTCGCCGACACCGTC

GTCAGGTTGAACCTCCAGAAGCTGGCCTGCATCACCGAAGCCATGGCGCGAGGGGAAGGT
GGCGGGGGCGGCACCGCCGCCGCCGGCGACACAAGTCATCATTGA
>itb09g28750.t1
ATGGAGAAAGCTGAGAGCTCGATGAACAGTGATTTGCATCGAGGAGAAGATGGAGAAGT
CGGGTCGGAGTCGACCCATCACTTGACTGTTCCGCCCGGATTGAACCCGGACGAGTTCGA
GGAGCTGAGGCCGTGGGCGATTGAGTTCCACACCTACCGAGTCAACTCGGGCCAATGCTC
CTCCCTCCTGGCGCAGCGAATCCACGCGCCGCCGGACACGGTGTGGTCCGTGGTTCGCCA
GTTCGACAAGCCGCACACCTACAAGCACTTCATCAAGAGCTGCACCGTGTGCGAGGGGTT
CCGGATGGCGGTGGGCGACACGCGCTACGTCAACGTCATCTCCGGCCTGCCGGCGGCCAC
CAGCACCGAGCGTCTCGACATCCTCGACGACGGCCGCCGCGTCACGGGCTTCAGCATCAT
TGGCGGCGAGCACCGCCTGAGGAACTACCGCTCCGTCACCTCCGTCCACGGCCTCCGCCG
CCGCGACGGCGGCCGGAGCTGCACCGCCGTGCTGGAATCCTACGTGGTGGACGTGCCGGA
GGGGAACACGGAGGAGGACACCAAGCTTTTCGCCGACACCGTCGTTAAGCTCAACCTTCA
AAAACTCGCATCCATCACCGAAGGAATCAATCAAGACAGAAACAACGATGCTAATGAAG
AAGATAGTGGTAATAATACAACCGAACGTCACACTTGA
>itb06g07990.t1
ATGGAGAAGACAGTCCAAATCCCTCAAGGGCTAACGGAAGAGGAATATTCCCAGCTGGA
AACGGTGATCCAATCTTACCACACCTTCGACCCCAGGCCCAACACGTGCACGTCCCTGATA
ACGCAGCACATCGACGCGCCGGCGAGCGTGGTGTGGCCGTTCGTCCGCCGCTTCGACAAC
CCGCAGAAGTACAAGCACTTCATCAAGAGCTGCCGGATGGTCTCCGGCGACGGCGGCGTG
GGGAGCGTCCGGGAAGTCACCGTGGTCTCCGGCATCCCCGCGTCCACCAGCACGGAGCGC
CTCGAGATCCTCGACGACGACAAGCGAATCATAAGCTTCCGAGTGGTCGGCGGCGAGCAC
CGGCTGAACAACTACCAGAGCGTCACTTCCGTCAACGAGTTCCAGAAGAAGAATGGGAGC
GTTTACACGGTGGTTTTGGAGTCGTATATCGTTGATATACCGGAAGGGAATACGGTGGTG
GACACAAAGATGTTCACCGATACCGTCGTCAAGCTCAACCTGCAGAAGCTTTGCATGGTG
GCAATGGCCGCTTTGCATGGCCACGAATAA
>itb15g21060.t1
ATGGCGGAGAGAGCCCAAATCCCGCAGGGGCTAACGGAGGAGGAATTCTCGGAGCTAGA
GCCGTTGATCCGGCGGTACCACACCTTCGATCACGTGCCCAACACATGCACGTCTCTGATC
ACGCAGCACATCGACGCGCCGGCGAGCGTGGTGTGGCCCATCGTCCGCCGCTTCGACAAC
CCGGAGAAGTACAAGCATTTCATCAAGAGCTGCCGGATGACCGGCGACGGCGGCGTGGG
GAGCATCCGGGAAGTCACCGTCGTCTCCGGCATCCCCGCCGCCACCAGCACCGAGCGCCT
CGAGATCCTCGACGACCACAACCACATCCTCAGCTTCCGCGTCGTCGGCGGCGAGCACCG
CCTCAATAACTACCGGAGCGTCACCTCGGTCAACCAATTCCCCCGCCGCCGCGGGGCCCA
CAACAATAACAACAACAATAACAATAATAATAACGGCTTTTACACAGTCGTTTTGGAGTC
TTATATTGTCGATATACCCGAAGGGAACACCGGAGCCGATACGCGTATGTTTACCGACAC
GGTGGTTAAGCTGAATCTGCAGAAGCTTGGGGTGGTGGCCATGGCGGCTTTGCATGGCGG
AGGCGGAGGCGGAGGTAGTGGTGGTCACTCATGA
>itb15g06930.t1
ATGTCTGAAATGCTTCCGAATCCGCCGGAGAGGTCTGTTTTGCTTCAAAGACTAATCCCGG
GGGCGGCCGCCACGTGTAAGCAATCGCAGCCGCAGTATCAACGGCGGACGCCGATTCCCT
GCGACAGCCATGTCCCGGAGACGGCGGCGCGGTATCACGCGTATTCGGTGAGTCCCAACC
AGTGCTGCTCCGCCGTGATCCAGTACATCGCGGCCCCGGTGTCCGCCGTGTGGGGCGTGGT
GCGGCGCTTCGATAACCCGCAGGCGTACAAGCACTTCGTGAAGAGCTGCCACGTCATCGC

GGGGGAGGGCGGCGTGGGGACGCTCCGCGAGGTGCGCGTGGTGTCCGGACTCCCCGCGG
CCACCAGCACGGAGCGGTTAGAGATCCTGGACGAGGAGCGCCACGTCATCAGCTTCAGCG
TCGTCGGCGGGGACCACCGCCTGGCCAACTACCGATCCGTCACGACGCTCCACGCGGGGG
CGGGCGGCGGAGACGGCGAGGACGAGGACGAGGACGGCACCGTCGTGGTGGAGTCGTAC
GTGGTCGACATCCCGCCGGGGAACACGCGGGAGGAGACGTGCGTGTTCGTCGACACTATC
GTGAAATGCAACCTCCAGTCGCTGGCGCAGATCGCCGAGAGCTCCGGCCGACGAAACGCG
GCTAGCTTGAATTGA
>itb12g11410.t1
ATGGCTCCCAACCCACCAAAGACTTCGGTTCTACTTCAAAGAATCAGCACTCGCAAGCAA
TCTCAACGGCGCTCATCGCATATAATATCTTGCGCCGCCGCGGCCTCGGAGGTCCCGGAA
ACGGTGGCCCGCTATCACGCCCATCCGGTGGGACCCAACCAGTGCTGCTCCGCCGTGATC
CAGCGGATCTCCGCCCCCGTCTCCACCGTTTGGTCCGTGGTCCGCCGCTTCGACAACCCGC
AGGCCTACAAGCACTTCGTGAAGAGCTGCGACGTCATCGTGGGGGACGGCGACGTGGGCA
CCCTCCGCGAGGTCCGCGTCATCTCCGGCCTCCCCGCCGGCAGCAGCACCGAGCGGCTCG
AGATCCTCGACGAGGAGCGCCACGTCATCAGCTTCAGCGTCGTCGACGGCGACCACCGCC
TCGCCAACTACCGCTCCGTCACCACCCTCCACGCGGACGTGGGCGGGACCACCACCGTCG
TGGAGTCCTACGTTGTTGATGTCCCGCCGGGAAACACGAGAGACGAAACCTGCGTCTTCG
TCGACACTATCGTCAAATGCAATCTCCAGTCGCTGGCGCAGATCGCCCAGAATCCGGCCG
ACGAAACAACAACTGAAGGGATCGGATCTAATTAA
>itb04g00250.t1
ATGGAAGCAGAGTACATAGAGAGATTCCACAAGCATCAGCCTACAGAGTATCAGTGTTCT
TCCATTGTTACCAACCACATCAAAGCTCCCACTGATATTGTTTGGTCACTGGTGAGGAGGT
TTGATCAGCCACAGAAGTATAAGCCATTTGTTAGCAGGTGTACAGTTTTGGGTGATCTTGC
AATTGGGAGTGTTAGAGAGGTAGATGTGAAGTCAGGACTTCCTGCTACAACCAGCACTGA
AAGGCTGGAACTTCTTGATGATGAGGAGCATATCCTTGGGATCAGGATTGTTGGTGGTGA
TCACAGACTGAAGAACTACTCTTCAGTCATTACAGTCCATCCAGAGATAATTGATGGGAG
ACCAGGAACACTGGTGATCGAGTCATTTTTGGTAGATGTGCCTGAAGGGAACACTCGAGA
CGATACCTGCTACTTTGTGAATGCACTTATCAACTGTAACCTGAAAGCTTTGGCAGATGTC
TCGGAGAGAATGGCCGTTCAGGATGGGAATGGGATTGCATCTACGACTGTGAGCTGGGCA
TCTAGTGCCTATATTAAGCCTTAA
>itb12g24010.t1
ATGCCTACTACAGTTCACTGCCATATATCCGATTCGCACCACCACCTCTCCGCCGCTGTCG
CGGCGGCGACGACGGCGGTGAGGATACAGAAGCAGCCGCCGTTGCCGACGTGGACGGTG
GTGGTCCCAGACAGCTTGCTGCAGTACCACGTCCACGCGGTGGGGCCCAACCAGTGCTGC
TCCGCGGTGGTGCAGGAGGTGTCCGCCCCGCTGGACGCCGTGTGGGCCCTGGTCCGCCGC
TTCGACAACCCGCAGGCCTACAAGCATTTCCTGAAGAGCTGCCACGTCATCGTGGGCGAC
GGCAGCAGCGTGGGCACGCTCCGCGAGGTGCGCGTGGTGTCCGGCGTCCCGGCCGTCTCC
AGCACCGAGCGCCTCGATATCCTCGACGACGAGCGACACGTGTTCGGCTTCAGCGTCGTC
GGCGGCGACCACCGCCTCCGCAACTACCGCTCCGTCACCACTCTCCACCACCGCGCCGCC
GCAGATTCCGAACACCACAAGACGGTCGTCGTGGAATCCTTCGTCGTCGACGTCCCGCCG
GGGAATACTAAGGAAGAGACGTGCGTCTTCGTTGACACCATCGTGCGGTGCAATCTCCAG
TCGTTAGCGCAAATCGCGGAAAGCTCCGGCAAGAATAATCAAAACATCGTCAAATCTTCT
TGTAATTAA
>itb03g15060.t1

ATGCCTTCTGCGCTTCAGCTTCATCAGCGCGGCTCGGCGGCGGCTGCGGCTGGAGTGGGG
AGTGTTTATAAGCAGGCGGCTCAGGCGGCGAGGTGGATGATTCCGGTGTCTATATCGGTG
CCGGAGCACGTGCTGCAGTACCACACGCACGCGGTGGGGGCGGGGCAGTGCTGCGCGGC
GGTGGTGCAGGAGGTGGCGGCGCCGCTGGAGGCGGTGTGGCGGCTGGTGCGCCGCTTCGA
CAAGCCGCAGGCGTACAAGCACTTCCTGAAGAGCTGCCACGTCATTGTCGGCGACGGGGA
CGTGGGCACGCTCCGCGAGGTGCGCGTGGTGTCCGGCCTCCCCGCCGCCTCCAGCACCGA
GCGCCTCGAGATCCTCGACGACGAGAAGCACGTGCTCAGCTTCGCCGTCGTCGGCGGCGA
TCACCGCCTCAACAACTACCGCTCCGTCACCACCCTCCACCCCCACCACCGCAACAACACC
ACCGTCGTCGTCGAGTCCTACGTCGTCGACGTCCCCCCCGGCAACACCAACGACGAAACC
TGCGTCTTCGTCGACACCATCGTACGTTGCAATCTTCAATCCCTAGCGCAAATCGCGGAAA
ACTCCAACAACTCTCCAAATCAAGAACTGAAAAGTCATACTACCAATACAGTGATAGATG
TTCGTTGA
>itb08g08610.t1
ATGACGGCCGACATGATGAGCGCCGATAGAAGAACCGACGTAGAGGATGAGTACATTAG
GAGGCACCACAAGCACGAGGTCAGGGAGAATCAGTGTAGTTCCTGTCTTGTAAAGCACAT
CAGAGCTCCAGTTCATCTTGTTTGGTCATTGGTAAGGAGGTTTGATCAGCCTCAGAGGTAC
AAACCGTTTGTGAGCAGGTGTATTGTGCAGGGCGACCTTGAAATTGGAAGTGTTAGAGAA
GTTAATGTTAAGTCTGGCCTTCCAGCCACCACCAGCAAGGAGAGGCTAGAGCTACTGGAT
GACGACGAGCACATATTTAGCATGAGGATTGTTGGTGGAGATCACAGGCTAAGGAACTAC
TCATCAATCGTGACTGTACACCCAGAGATCATTGATGGCAGGCCTGGAACATTGGTCATA
GAATCATTTGTGGTGGACATCCCAGACGGGAACACCAAGGATGAAACATGCTACTTCGTG
GAGGCGCTGATTAAGTGCAACCTCAAGTCATTGGCTGATGTCTCGGAGCGATTGGCAGTA
CAGGGCCGCACGGAGCCTATTGACAGAGTGTAG
>itb03g23630.t1
ATGAACGCGAACGGATACAGCAGTGTTGAAAGAGAGTACATAAGGAAACATCATAGGCA
AGAGCCGGCGGGGAATCAATGCAGTTCGTTTCTGATCAAGCACATTAGAGCGCCTGTTCA
TCTCGTTTGGTCATTGGTTCGGAGGTTTGATCAACCACAGAAGTACAAGCCCTTTGTCAGC
CGGTGCATAGCACAGGGAAACCTTGAGATTGGTAGTCTTAGAGAAGTTGATGTCAAATCA
GGCCTTCCTGCCACAACAAGCACGGAAAGGTTAGAACTTCTGGATGATGATGAACATATC
CTCAGCGTGAAGATTGTTGGGGGAGATCACAGACTTAGGAACTACTCCTCTGTCATATCTG
TCCATCCAGAAGTAATTGATGGACGACCCGGAACTCTGGTTATTGAATCATTTGTGGTAGA
TGTGCCTGACGGAAACACTAAAGATGAGACCTGCTACTTTGTTGAAGCATTGATCAAGTG
CAATCTGAAATCTCTAGCCGATGTTTCCGAGAGGCTTGCTTTGCAGGGCAGGACAGAGCC
CATTGATCAGGTGTAG
>itb05g20040.t1
ATGAGGGTAGGTGAGACTCCGGCGACGGAAGTGAAGATGAAGGAAGATGGATACAGCGG
CAGTGAAAGAGAGTACATAAGGAAGCACCATAGGCACCAGCCGGCAGAGAATCAATGCA
CTTCCTTTCTGATCAAGCACATCAGAGCACCTCTTCATCTCGTGTGGTCATTGGTTAGGAG
ATTTGATGAACCACAGACGTACAAGCCATTTGTGAGGAGGTGCATTGTGCATGGAAACGT
TGAGATTGGGAGTGTTAGAGAAGTTGATGTGAGATCAGGGCTTCCCGCCACCACAAGCAC
TGAAAGATTGGAGCTCCTTGATGACGACCAGCATATCCTCAGATTCAGGATTGTTGGGGG
AGATCACAGGCTCACGAACTACTCTTCCATTCTCTCTCTCCATCCTGAGGTGGTTGATGGA
AGACCTGCAACTCTGGTGATCGAGTCGTTTGTGGTAGATGTACCTCCGGGGAACACTAAA
GACGAAACCTGCTACTTTGTACAAGCATTCATCAACTGCAATCTCAGATCTCTTGCTCACG

CTTCAGAGCAGCTATCTGAGACAACCCACTCATCATGTGTAATGGTCCAGGCAGACTCTGT
GGAACCACATCTAAGTTGCTGTTGA
>itb12g26370.t2ATGAACGCGGCTGGGAACGGCGGTGTGGAGAGAGAGTACATAACGAAAC
ATCACAGGCACGCGCCGGCGGAGAATCAATGCAGTTCGTTTCTGATCAAGCACATCAGAG
CACCTGTTAACCTCGTTTGGTCATTGGTTAGGAGGTTTGATCAGCCACAGAAATACAAGCC
CTTTATCAGCAGGTGCATTAGTCAGGGAAGTCTTGAGATTGGTAGTCTTAGAGAAGTTGAT
GTCAAGTCGGGCCTGCCTGCCACAACAAGCACAGAAAGGTTGGAGCTTCTCGATGATGAT
GAGCATATACTCAGTGTGAGGATTGTTGGTGGGGATCACCGACTTCGGAACTATTCCTCAG
TCATATCTGTCCATCCAGAGGTCATTGACGGAAGACCTGGAACTGTGGTGATAGAATCATT
CGTGGTAGATGTGCCAGAAGGAAACACTATCGACGAAACCTGCTACTTTGTTGAAGCATT
AATCAAATGCAATCTGAAATCTCTTGCGGATGTTTCAGAGCGGCTTGCTGTGCAGGGCAG
GACAGAACCCATAGATCCTATGTAA
>itb12g26370.t1
ATGAACGCGGCTGGGAACGGCGGTGTGGAGAGAGAGTACATAACGAAACATCACAGGCA
CGCGCCGGCGGAGAATCAATGCAGTTCGTTTCTGATCAAGCACATCAGAGCACCTGTTAA
CCTCGTTTGGTCATTGGTTAGGAGGTTTGATCAGCCACAGAAATACAAGCCCTTTATCAGC
AGGTGCATTAGTCAGGGAAGTCTTGAGATTGGTAGTCTTAGAGAAGTTGATGTCAAGTCG
GGCCTGCCTGCCACAACAAGCACAGAAAGGTTGGAGCTTCTCGATGATGATGAGCATATA
CTCAGTGTGAGGATTGTTGGTGGGGATCACCGACTTCGGAACTATTCCTCAGTCATATCTG
TCCATCCAGAGGTCATTGACGGAAGACCTGGAACTGTGGTGATAGAATCATTCGTGGTAG
ATGTGCCAGAAGGAAACACTATCGACGAAACCTGCTACTTTGTTGAAGCATTAATCAAAT
GCAATCTGAAATCTCTTGCGGATGTTTCAGAGCGGCTTGCTGTGCAGGGCAGGACAGAAC
CCATAGATCCTATGTAA
>itb12g18500.t1
ATGACGGTCAGCATGCTCGGCGGCGATAGGAGGACCGGTGTGGAGGACGAGTACATCCG
GAGACACCACAGACATGAGGTCAGAGACAACCAGTGTAGCTCCTCGCTCGTTAAGCACAT
CAGAGCTCCGGTTCATCTCGTATGGTCGTTAGTGAGGAGATTTGATCAACCACAAAGGTAT
AAACCATTTGTGAGCAGGTGCATCGTGCAGGGAGACCTTGAAATTGGAAGTGTTAGAGAA
GTTAATGTTAGGTCAGGACTTCCGGCAACCACAAGCAAGGAGAGACTAGAGCTCCTAGAT
GATAATGAACATATCTTTAGCATGAGGATTGTTGGTGGCGATCACAGGCTAAGGAATTAC
TCATCAATAATCACCGTTCATCCAGAGGTCATCGACAGCAGGCCTGGGACATTGGTAATT
GAGTCATTTGTCGTAGACGTTCCTGATGGGAACACTCAGGATGAAACGTGCTACTTTGTGG
AGGCTCTGATCAAGTGCAATCTCAAGTCTCTGGCTGAGGTCTCGGAACGACTGGCAGTTC
AGGGCCATACAGAGCCCATCGACAGAATCTGA

Amino acid sequences

>IbPYL1
MRVGETPATEVKMKEDGYSGSEREYIRKHHRHQPAENQCTSFLIKHIRAPLHLVWSLVRRFDEP
QTYKPFVRRCIVHGNVEIGSVREVDVRSGLPATTSTERLELLDDDQHILRFRIVGGDHRLTNYSS
ILSLHPEVVDGRPATLVIESFVVDVPPGNTKDETCYFVQAFINCNLRSLAHASEQLSETTHSSCV
MVQADSMEPHLSCC
>IbPYL2
MVLEEKMEAEYIERFHKHQPTEYQCSSIVTNHIKAPTDIVWSLVRRFDQPQKYKPFVSRCTVLG

DLAIGSVREVDVKSGLPATTSTERLELLDDEEHILGIRIVGGDHRLKNYSSVITVHPEIIDGRPGT
LVIEVIFDVSERMAIQDGNGIASTTVSWASSAYIKP
>IbPYL3
MSSDPNQYGSSTTHHLTVPPGLGPEEFEELKPWITQFHSYRVNSGKCSSLLAQRVHAPPDTVWS
VVRRFDRPQTYKHFIKSCSVGEGFRMAVGDTRDVNVISGLPAATSTERLDVLDEDRRVTGFSII
GGEHRLRNYRSVTSVHGMSSSPRHPTTVVLESYVVDVPEGNTEEDTKLFADTVVRLNLQKLA
CITEAMAGRWRRHRRRRRRHKSSLRVR
>IbPYL4
MLGGDRRTGVEDEYIRRHHRHEVRDNQCSSSLVKHIRAPVHLVSLLRTLLFCSSDFLFVSAQYY
SDLLVPATDCDPFEFTPLTMDVWIFDEVWSLVRRFDQPQRYKPFVSRCIVQGDLEIGSVREVNV
RSGLPATTSKERLELLDDNEHIFSMRIVGGDHRLRNYSSIITVHPEIIDSRPGTLVIESFVVDVPDG
NTQDETCYFVEALIRCNLKSLAEVSERLAVQGHTEPIDRI
>IbPYL5
MPTTVHCHISDSHHHLSAAVSAATTAVRIQKQSPLPTWTVVVPDSLLQYHVHAVGPNQCCSAV
VQEVSAPLDAVWALVRRFDNPQAYKHFLKSCHVIVGDGSSVGTLREVRVVSGVPAVSSTERLDI
LDDERHVFGFSVVGGDHRLRNYRSVTTLHHRAAADSEHHKTVVVESFVVDVPPGNTKEETCV
FVDTIVRCNLQSLAQIAENSGKNNQNIVKSSCN
>IbPYL6
MNAAGNGGVEREYITKHHRHAPAENQCSSFLIKHIRAPVNLVWSLVRRFDQPQKYKPFISRCIS
QGSLEIGSLREVDVKSGLPATTSTERLELLDDDEHILSVRIVGGDHRLRNYSSVISVHPEVIDGRP
GTVVIESFVVDVPEGNTIDETCYFVEALIKCNLKSLADVSERLAVQGRTEPIDPV
>IbPYL7
MSEMLPNPPERSVLLQRLIPGAGATCKQSQPQYQRRTPIPCDSHVPETAARYHAYSVSPNQCCS
AVIQYIAAPVSAVWGVVRRFDNPQAYKHFVKSCHVIAGEGDVGTLREVRVVSGLPAATSTERL
EILDEERHVISFSVVGGDHRLANYRSVTTLHAGAGGGDGEEEDEDGTVVVESYVVDIPPGNTR
EETCVFVDTIVKCNLQSLAQIAESSGRRNSASLN
>IbPYL8
MPSALQLHRSNQLGSAAVAAASVYKQAAAAQAARWMIPVSISVPEHVLQYHTHAVGAGQCC
AAVVQEVAAPLEAVWRLVRRFDKPQAYKHFLKSCHVIVGDGDVGTLREVRVVSGLPAASSTER
LEILDDEKHVLSFAVVGGDHRLNNYRSVTTLHPTTATTPPSSSSPTSSTSPPATPTTKPASSSTPSS
RTEKSYYQYSDRCSLKIIKLRFVIRFGGCDEDLV
>IbPYL9
MNANGYSSVEREYIRKHHRQEPAGNQCSSFLIKHIRAPVHLVWSLVRRFDQPQKYKPFVSRCIA
QGNLEIGSLREVDVKSGLPATTSTERLELLDDDEHILSVKIVGGDHRLRNYSSVISVHPEVIDGR
PGTLVIESFVVDVPEGNTKDETCYFVEALIKCNLKSLADVSERLALQGRTEPIDQV
>IbPYL10
MTADMMSADRRTDVEDEYIRRHHKHEVRENQCSSCLVKHIRAPVHLVWSLVRRFDQPQRYKP
FVSRCIVQGDLEIGSVREVNVKSGLPATTSKERLELLDDDEHIFSMRIVGGDHRLRFNYLILVDV
VCSVLHLPEHFGLLGCGCSLKWQCGWLRNLICYFLFVLLARKDYRRISQAVLKFHPESQLLILS
HPAFKNMQNYSSIVTVHPEIIDGRPGTLVIESFVVDIPDGNTKDETCYCGGAD
>IbPYL11
MEKAESSMNSDLQRGEDGEVGSESTHHLTVPPGLNPDEFEELRPWAIEFHTYRVNSGQCSSLL
AQRIHAPPDTVWSVVRQFDKPHTYKHFIKSCTVCEGFRMAVGDTRYVNVISGLPAATSTERLDI
LDDGRRVTGFSIIGGEHRLRNYRSVTSVHGLRRRDGGRSCTAVLESYVVDVPEGNTEEDTKLFA

DTVVKLNLQKLASITEGINQDRNNDANEEDSGNNTTERHT

>IbPYL12L

MAGHQKIEVDVETIEVLEGDGKAIGSIRLIKYGEGAPLLTFAKEKIDAVDDEKKTVSYHVLEGD
ILKHYKHFKAFLCVTPKGDGSLVKWWCEFDKASPEVPEPHFIRDAAVKTFKDLEAFLKA

>IbPYL13L

MLLPAGAANPRAAGHGVVRGSPVRQAAHLQALHQELHRVRGFRMAVGDTRYVNVISGLPAA
TSTERLDILDDGRRVTGFSIIGGEHRLRNYRSVTSVHGLRRRDVGRAAPPCWNPTWWTCRRNT
EEDTKLFADTVVKLNLQKLASITEGINQDRNNDANEEDSGNGDKEMLAWSDHFVQVLNNND
VGMMVL

>ItfPYL1

MSSDPNQYGSSTTHHLTVPPGLGPEEFEELKPWITQFHSYRVNSGKCSSLLAQRVHAPPDTVWS
VVRRFDRPQTYKHFIKSCSVGEGFRMAVGDTRDVNVISGLPAATSTERLDVLDEDRRVTGFSII
GGEHRLRNYRSVTSVHGLSSSPRHPTTVVLESYVVDVPEGNTEEDTKLFADTVVRLNLQKLAC
ITEAMARGEGGGGATAAAAGDTSHH

>ItfPYL2

MPSALQLHRSNQLGSAAAAAATSVYKQAAAAQAARWMIPVSISVPENVLQYHTHAVGAGQC
CAAVVQEVAAPLEAVWRLVRRFDKPQAYKHFLKSCHVIVGDGDVGTLREVRVVSGLPAASSTE
RLEILDDEKHVLSFAVVGGDHRLNNYRSVTTLHPHHRNNTTVVVESYVVDVPPGNTNDETCV
FVDTIVRCNLQSLAQIAENSNNSPNQELKSHTTNTVIDVR

>ItfPYL3

MFLYRNGFGGVVLNREREATMNANGYSSVEREYIRKHHRQEPAGNQCSSFLIKHIRAPVHLVW
SLVRRFDQPQKYKPFVSRCIAQGNLEIGSLREVDVKSGLPATTSTERLELLDDDEHILSVKIVGG
DHRLRNYSSVISVHPEVIDGRPGTLVIESFVVDVPEGNTKDETCYFVEALIKCNLKSLADVSERL
ALQGRTEPIDQV

>ItfPYL4

MVLEEKMEAEYIERFHKHQPTEYQCSSIVTNHIKAPTDIVWSLVRRFDQPQKYKPFVSRCTVLG
DLAIGSVREVDVKSGLPATTSTERLELLDDEEHILGIRIVGGDHRLKNYSSVITVHPEIIDGRPGT
LVIESFLVDVPEGNTRDDTCYFVNALINCNLKALADVSERMAIQDGNGIASTTVSWASSAYIKP

>ItfPYL5

MEQRLIVPLNEQSGAGSRCMRTATLQLSLRSLLTSKASTARRLSRVSEWYALVAWDLWLLKLD
NLGFFKLHGLVTTRRKMMADTSSMAKQGPVLDVKPVKDDDGGYASGGWKSEDGRLSCGYSS
FRGKRASMEDFYDIKTSKIDGKTICLFGIFDGHGGSRAAEYLKQNLFENLMKHPEFVTNTKHAI
SETYQQTDRDFLESEKDSLRDDGSTASTAVLVGNHLYVANVGDSRTIVSKKGKAIPLSQDHKPN
RSDERKRIENAGGVIMWAGTWRVGGVLAMSRAFGNRMLKQYVVAEPEIQEEVIDEELELLVL
ASDGLWDVVPNEDAVSLAESEESPEAAARKLTETAFTRGSADNITCIVVKFHHKKAEPEWEWE
WEWEMRVGETPATEVKMKEDGYSGSEREYIRKHHRHQPAENQCTSFLIKHIRAPLHLVWSLVR
RFDEPQTYKPFVRRCIVHGNVEIGSVREVDVRSGLPATTSTERLELLDDDQHILRFRIVGGDHRL
TNYSSILSLHPEVVDGRPATLVIESFVVDVPPGNTKDETCYFVQAFINCNLRSLAHASEQLSETT
HSSCVMVQPDSMEPHLSCC

>ItfPYL6

MEKTVQIPQGLTEEEYSQLETVIQSYHTFDPRPNTCTSLITQHIDAPASVVWPFVRRFDNPQKY
KHFIKSCRMVSGDGGVGSVREVTVVSGIPASTSTERLEILDDDKRIISFRVVGGEHRLNNYQSV
TSVNEFQKKNGSVYTVVLESYIVDIPEGNTVVDTKMFTDTVVKLNLQKLCMV
AMAALHGHE

>ItfPYL7
MTADMMSADRRTDVEDEYIRRHHKHEVRENQCSSCLVKHIRAPVHLVWSLVRRFDQPQRYKP
FVSRCIVQGDLEIGSVREVNVKSGLPATTSKERLELLDDDEHIFSMRIVGGDHRLRNYSSIVTVH
PEIIDGRPGTLVIESFVVDIPDGNTKDETCYFVEALIKCNLKSLADVSERLAVQGRTEPIDRV
>ItfPYL8
MPPTIVTVELRIHAPPDTVWSVVRQFDKPHTYKHFIKSCTVCEGFRMAVGDTRYVNVISGLPAA
TSTERLDILDDGRRVTGFSIIGGEHRLRNYRSVTSVHGLRRRDGGRSCTAVLESYVVDVPEGNT
EEDTKLFADTVVKLNLQKLASITEGINQDRNNDANEEDSGNNTTERHT
>ItfPYL9
MAPNPPKTSVLLQRISTRKQSQRRSSPIISCAAAASEVPEAVARYHAHPVGPNQCCSAVIQRISAP
VSTVWSVVRRFDNPQAYKHFVKSCDVIVGDGDVGTLREVRVISGLPAGSSTERLEILDEERHVI
SFSVVDGDHRLANYRSVTTLHADAGGTTTVVESYVVDVPPGNTRDETCVFVDTIVKCNLQSL
AQIAQNSGRRNNN
>ItfPYL10
MTVSMLGGDRRTGVEDEYIRRHHRHEVRDNQCSSSLVKHIRAPVHLVWSLVRRFDQPQRYKP
FVSRCIVQGDLEIGSVREVNVRSGLPATTSKERLELLDDNEHIFSMRIVGGDHRLRNYSSIITVHP
EVIDSRPGTLVIESFVVDVPDGNTQDETCYFVEALIKCNLKSLAEVSERLAVQGHTEPIDRI
>ItfPYL11
MPTTVHCHISDSHHHLSAAVAAATTAVRIQKQPPLPTWTVVVPDSLLQYHVHAVGPNQCCSAV
VQEVSAPLDAVWALVRRFDNPQAYKHFLKSCHVIVGDGSSVGTLREVRVVSGVPAVSSTERLDI
LDDERHVFGFSVVGGDHRLRNYRSVTTLHHRAADDHEHNKTVVVESFVVDVPPGNTKEETC
VFVDTIVRCNLQSLAQIAENSGKNNQNIVKSSCN
>ItfPYL12
MNAAGNGGVEREYITKHHRHAPAENQCSSFLIKHIRAPVNLVWSLVRRFDQPQKYKPFISRCIS
QGSLEIGSLREVDVKSGLPATTSTERLELLDDDEHILSVRIVGGDHRLRNYASVISVHPEVIDGRP
GTVVIESFVVDVPEGNTIDETCYFVEALIKCNLKSLADVSERLAVQGRTEPIDPV
>ItfPYL13
MSEMLPNPPERSVLLQRLIPGAGATCKQSQPQYQRRTPIPCDSHVPETAARYHAYSVSPNQCCS
AVIQYIAAPVSAVWGVVRRFDNPQAYKHFVKSCHVIAGEGDVGTLREVRVVSGLPAATSTERL
EILDEERHVISFSVVGGDHRLANYRSVTTLHAGAGGGDGEDEDGTVVVESYVVDIPPGNTREE
TCVFVDTIVKCNLQSLAQIAESSGRRNAASLN
>ItfPYL14
MAERVQIPQGLTEEEFSELEPLIRRYHTFDHVPNTCTSLITQHIDAPASVVWPIVRRFDNPEKYK
HFIKSCRMTGDGGVGSIREVTVVSGIPAATSTERLEILDDHNHILSFRVVGGEHRLNNYRSVTSV
NQFPRRRGAHNNNNNNNDFYTVVLESYIVDIPEGNTGADTRMFTDTVVKL
NLQKLGVVAMAALHGGGGGGGSGGHS
>ItbPYL1
MPSALQLHQRGSAAAAAGVGSVYKQAAQAARWMIPVSISVPEHVLQYHTHAVGAGQCCAAV
VQEVAAPLEAVWRLVRRFDKPQAYKHFLKSCHVIVGDGDVGTLREVRVVSGLPAASSTERLEI
LDDEKHVLSFAVVGGDHRLNNYRSVTTLHPHHRNNTTVVVESYVVDVPPGNTNDETCVFVDT
IVRCNLQSLAQIAENSNNSPNQELKSHTTNTVIDVR
>ItbPYL2
MNANGYSSVEREYIRKHHRQEPAGNQCSSFLIKHIRAPVHLVWSLVRRFDQPQKYKPFVSRCIA
QGNLEIGSLREVDVKSGLPATTSTERLELLDDDEHILSVKIVGGDHRLRNYSSVISVHPEVIDGR

PGTLVIESFVVDVPDGNTKDETCYFVEALIKCNLKSLADVSERLALQGRTEPIDQV
>ItbPYL3
MEAEYIERFHKHQPTEYQCSSIVTNHIKAPTDIVWSLVRRFDQPQKYKPFVSRCTVLGDLAIGS
VREVDVKSGLPATTSTERLELLDDEEHILGIRIVGGDHRLKNYSSVITVHPEIIDGRPGTLVIESFL
VDVPEGNTRDDTCYFVNALINCNLKALADVSERMAVQDGNGIASTTVSWASSAYIKP
>ItbPYL4
MRVGETPATEVKMKEDGYSGSEREYIRKHHRHQPAENQCTSFLIKHIRAPLHLVWSLVRRFDEP
QTYKPFVRRCIVHGNVEIGSVREVDVRSGLPATTSTERLELLDDDQHILRFRIVGGDHRLTNYSS
ILSLHPEVVDGRPATLVIESFVVDVPPGNTKDETCYFVQAFINCNLRSLAHASEQLSETTHSSCV
MVQADSVEPHLSCC
>ItbPYL5
MEKTVQIPQGLTEEEYSQLETVIQSYHTFDPRPNTCTSLITQHIDAPASVVWPFVRRFDNPQKY
KHFIKSCRMVSGDGGVGSVREVTVVSGIPASTSTERLEILDDDKRIISFRVVGGEHRLNNYQSV
TSVNEFQKKNGSVYTVVLESYIVDIPEGNTVVDTKMFTDTVVKLNLQKLCMVAMAALHGHE
>ItbPYL6
MTADMMSADRRTDVEDEYIRRHHKHEVRENQCSSCLVKHIRAPVHLVWSLVRRFDQPQRYKP
FVSRCIVQGDLEIGSVREVNVKSGLPATTSKERLELLDDDEHIFSMRIVGGDHRLRNYSSIVTVH
PEIIDGRPGTLVIESFVVDIPDGNTKDETCYFVEALIKCNLKSLADVSERLAVQGRTEPIDRV
>ItbPYL7
MEKAESSMNSDLHRGEDGEVGSESTHHLTVPPGLNPDEFEELRPWAIEFHTYRVNSGQCSSLL
AQRIHAPPDTVWSVVRQFDKPHTYKHFIKSCTVCEGFRMAVGDTRYVNVISGLPAATSTERLDI
LDDGRRVTGFSIIGGEHRLRNYRSVTSVHGLRRRDGGRSCTAVLESYVVDVPEGNTEEDTKLFA
DTVVKLNLQKLASITEGINQDRNNDANEEDSGNNTTERHT
>ItbPYL8
MAPNPPKTSVLLQRISTRKQSQRRSSHIISCAAAASEVPETVARYHAHPVGPNQCCSAVIQRISA
PVSTVWSVVRRFDNPQAYKHFVKSCDVIVGDGDVGTLREVRVISGLPAGSSTERLEILDEERHV
ISFSVVDGDHRLANYRSVTTLHADVGGTTTVVESYVVDVPPGNTRDETCVFVDTIVKCNLQSL
AQIAQNPADETTTEGIGSN
>ItbPYL9
MTVSMLGGDRRTGVEDEYIRRHHRHEVRDNQCSSSLVKHIRAPVHLVWSLVRRFDQPQRYKP
FVSRCIVQGDLEIGSVREVNVRSGLPATTSKERLELLDDNEHIFSMRIVGGDHRLRNYSSIITVHP
EVIDSRPGTLVIESFVVDVPDGNTQDETCYFVEALIKCNLKSLAEVSERLAVQGHTEPIDRI
>ItbPYL10
MPTTVHCHISDSHHHLSAAVAAATTAVRIQKQPPLPTWTVVVPDSLLQYHVHAVGPNQCCSAV
VQEVSAPLDAVWALVRRFDNPQAYKHFLKSCHVIVGDGSSVGTLREVRVVSGVPAVSSTERLDI
LDDERHVFGFSVVGGDHRLRNYRSVTTLHHRAAADSEHHKTVVVESFVVDVPPGNTKEETCV
FVDTIVRCNLQSLAQIAESSGKNNQNIVKSSCN
>ItbPYL11.1
MNAAGNGGVEREYITKHHRHAPAENQCSSFLIKHIRAPVNLVWSLVRRFDQPQKYKPFISRCIS
QGSLEIGSLREVDVKSGLPATTSTERLELLDDDEHILSVRIVGGDHRLRNYSSVISVHPEVIDGRP
GTVVIESFVVDVPEGNTIDETCYFVEALIKCNLKSLADVSERLAVQGRTEPIDPM
>ItbPYL12
MSSNPNQYGSSSTHHLTVPPGLGPEEFEELKPWITQFHSYRVNSGKCSSLLAQRVHAPPDTVWS
VVRRFDRPQTYKHFIKSCSVGEGFRMAVGDTRDVNVISGLPAATSTERLDVLDEDRRVTGFSII

GGEHRLRNYRSVTSVHGLSSSPRHPTTVVLESYVVDVPEGNTEEDTKLFADTVVRLNLQKLAC
ITEAMARGEGGGGGTAAAAGDTSHH
>ItbPYL13
MSEMLPNPPERSVLLQRLIPGAAATCKQSQPQYQRRTPIPCDSHVPETAARYHAYSVSPNQCCS
AVIQYIAAPVSAVWGVVRRFDNPQAYKHFVKSCHVIAGEGGVGTLREVRVVSGLPAATSTERL
EILDEERHVISFSVVGGDHRLANYRSVTTLHAGAGGGDGEDEDEDGTVVVESYVVDIPPGNTR
EETCVFVDTIVKCNLQSLAQIAESSGRRNAASLN
>ItbPYL14
MAERAQIPQGLTEEEFSELEPLIRRYHTFDHVPNTCTSLITQHIDAPASVVWPIVRRFDNPEKYK
HFIKSCRMTGDGGVGSIREVTVVSGIPAATSTERLEILDDHNHILSFRVVGGEHRLNNYRSVTSV
NQFPRRRGAHNNNNNNNNNNNGFYTVVLESYIVDIPEGNTGADTRMFTDTVVKLNLQKLGV
VAMAALHGGGGGGSGGHS

Table S3. Specific primer sequences used for qRT-PCR analysis.

Primer code	Primer sequences (5' →3')	Application
IbARF-Q-F	CTTTGCCAAGAAGGAGATGC	Internal standard gene for qRT-PCR analysis
IbARF-Q-R	CTTGTCCTGACCACCAACA	
IbPYL1-Q-F	GATCTCTTGCTCACGCTTCAGA	qRT-PCR analysis
IbPYL1-Q-R	AGCAACTTAGATGTGGTTCCATAGAG	
IbPYL2-Q-F	CAGGATGGGAATGGGATTGC	
IbPYL2-Q-R	TTCAAGCAGTCTTCCCAATCTCA	
IbPYL3-Q-F	CTCTATGAGCAGTGACCCGAACC	
IbPYL3-Q-R	GGTACGAGTGGAACTGGGTGATC	
IbPYL4-Q-F	AGGTCTCAGAACGATTGGCAGT	
IbPYL4-Q-R	GTCACTCTACTCGCCTTCTATTGG	
IbPYL5-Q-F	CGGGGAATACTAAGGAAGAGAC	
IbPYL5-Q-R	ACGATGTTTTGATTATTCTTGCC	
IbPYL6-Q-F	CCATAGATCCTGTGTAAAGAGCCA	
IbPYL6-Q-R	GGAAGAGTAGCTATGAATGGTGTACC	
IbPYL7-Q-F	ACCGTCGTGGTGGAGTCGTA	
IbPYL7-Q-R	TCAATTCAAGCTAGCCGAGTTTC	
IbPYL8-Q-F	CAGTGATAGATGTTCGTTGAAGATTA	
IbPYL8-Q-R	GTTTGGTTACAAAACATCATACGAG	
IbPYL9-Q-F	TCCGAGAGGCTTGCTTTGC	
IbPYL9-Q-R	GCGTATATTCAAAAGCCCCAAGT	
IbPYL10-Q-F	CATCCCGAGTCCCAACTTCTAAT	
IbPYL10-Q-R	TCATCCTTGGTGTTCCCGTCT	
IbPYL11-Q-F	ACCTTCAAAAACTCGCATCCAT	
IbPYL11-Q-R	CGTTCGGTTGTATTATTACCACTATC	
